# Gene-activated matrices as nanomedicine platforms for bone regeneration: Delivery, translation and patent landscape

**DOI:** 10.1016/j.mtbio.2026.103808

**Published:** 2026-11-03

**Authors:** Hiba Abdelnabi, Salem Mohammed Alkaabi, Khaled Saeed Alderei, Ahmad Sultan Alaryani, Sahar Mohsin

**Affiliations:** Department of Anatomy, College of Medicine and Health Sciences, United Arab Emirates University UAEU, P.O. Box 15551, Al Ain, United Arab Emirates

**Keywords:** Gene-activated matrices, Bone regeneration, Biomaterial scaffolds, Nonviral gene delivery, CRISPR activation, Bone tissue engineering, Patent landscape

## Abstract

Protein biologics are constrained in critical-size bone defects not by insufficient potency, but by rapid clearance and the inability to sustain therapeutic signaling at the repair site. Dose escalation does not overcome this pharmacokinetic limitation and instead increases the risk of ectopic bone formation, osteolysis, and soft tissue toxicity. Sustained, localized delivery, not higher dosing, is the unmet requirement. Gene-activated matrices (GAM) address this problem by integrating nucleic acid cargo and delivery vectors within an implantable scaffold, converting infiltrating host cells into local sources of regenerative signals.

This review examines GAMs as nanomedicine platforms for bone regeneration, tracing their development from scaffold bioactivity and the limitation of protein biologics to nucleic acid-based delivery. We compare viral vectors, lipid nanoparticles, polymeric polyplexes, calcium phosphate nanoparticles, and scaffold-integrated systems across transduction efficiency, expression kinetics, immunogenicity, and bone-site retention. We then examine how scaffold composition, architecture, and fabrication govern vector retention, release, and substrate-mediated transfection. This review then evaluates nucleic acid cargo selection and coordinated osteogenic and angiogenic gene delivery, including pDNA, mRNA, siRNA, miRNA, and explores emerging multi-factor, stimuli-responsive, and CRISPR-based GAMs alongside their translational constraints.

A distinct feature of this review is its claim-level patent analysis, which identifies substantial intellectual-property white space in GAM development. Despite strong preclinical evidence, no GAM platform has simultaneously achieved scalable manufacturing, regulatory readiness as a combination product, and validation in a human-scale bone defect model. Clinical translation will require coordinated optimization of vector-scaffold interactions, manufacturing scalability, safety, and clinically relevant validation.

## Graphical abstract

**Figure:**
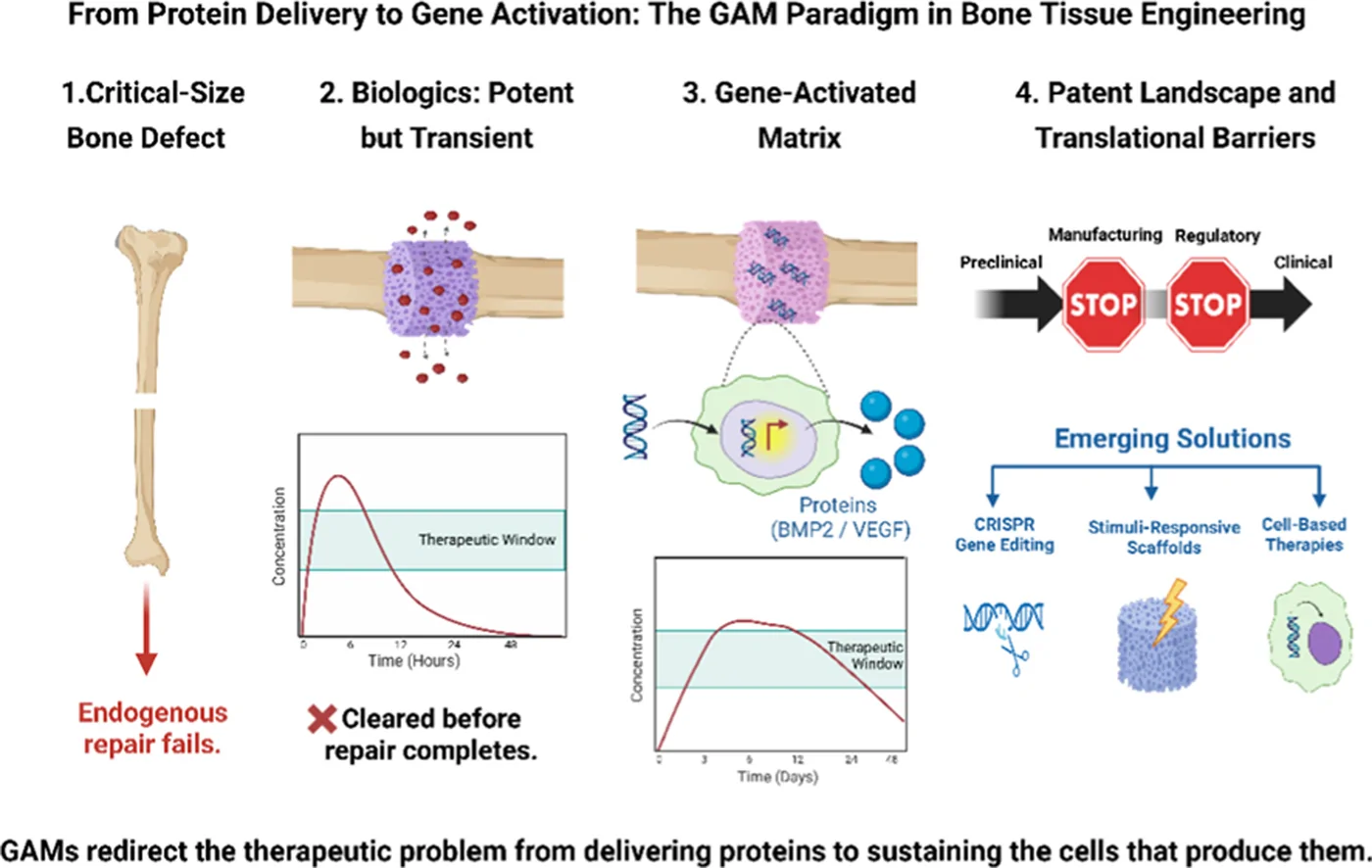

## 1 Background

Critical-size bone defects expose a mismatch between the biological requirements of bone regeneration and the duration of therapeutic signaling achievable at repair site. Bone is one of the few tissues capable of scarless regeneration through coordinated osteoblast- and osteoclast-mediated remodeling [1]. Angiogenesis is coupled to this process from the outset, supplying the oxygen and nutrients through early vascular invasion [2]. This regenerative capacity is limited. Defects exceeding a critical size, commonly defined as a loss of at least half the bone circumference over a length greater than 1 to 2 cm, exceed what endogenous signaling can bridge, and spontaneous repair arrests [3]. This threshold is shaped by species, anatomical site, age, periosteal integrity, and fixation method [4]. The result is a non-union that neither resolves nor progresses without external intervention.

Autografts remain the clinical benchmark, combining osteogenic cells, osteoinductive signals, and an osteoconductive matrix in a single immune-tolerated construct [5], [6]. Harvest volume is finite; donor site morbidity is a documented burden [5]. Allografts and xenografts expand supply but at a biological cost. Processing and sterilization eliminate viable osteogenic cells and reduce osteoinductive activity, leaving a graft that conducts but does not instruct [6], [7]. None of these strategies regenerate on their own; each still depends on the host to supply the biological activity the graft material can no longer generate. Fig. 1 compares autograft, bioactive scaffold, protein biologics, and GAM.

**Fig. 1:**
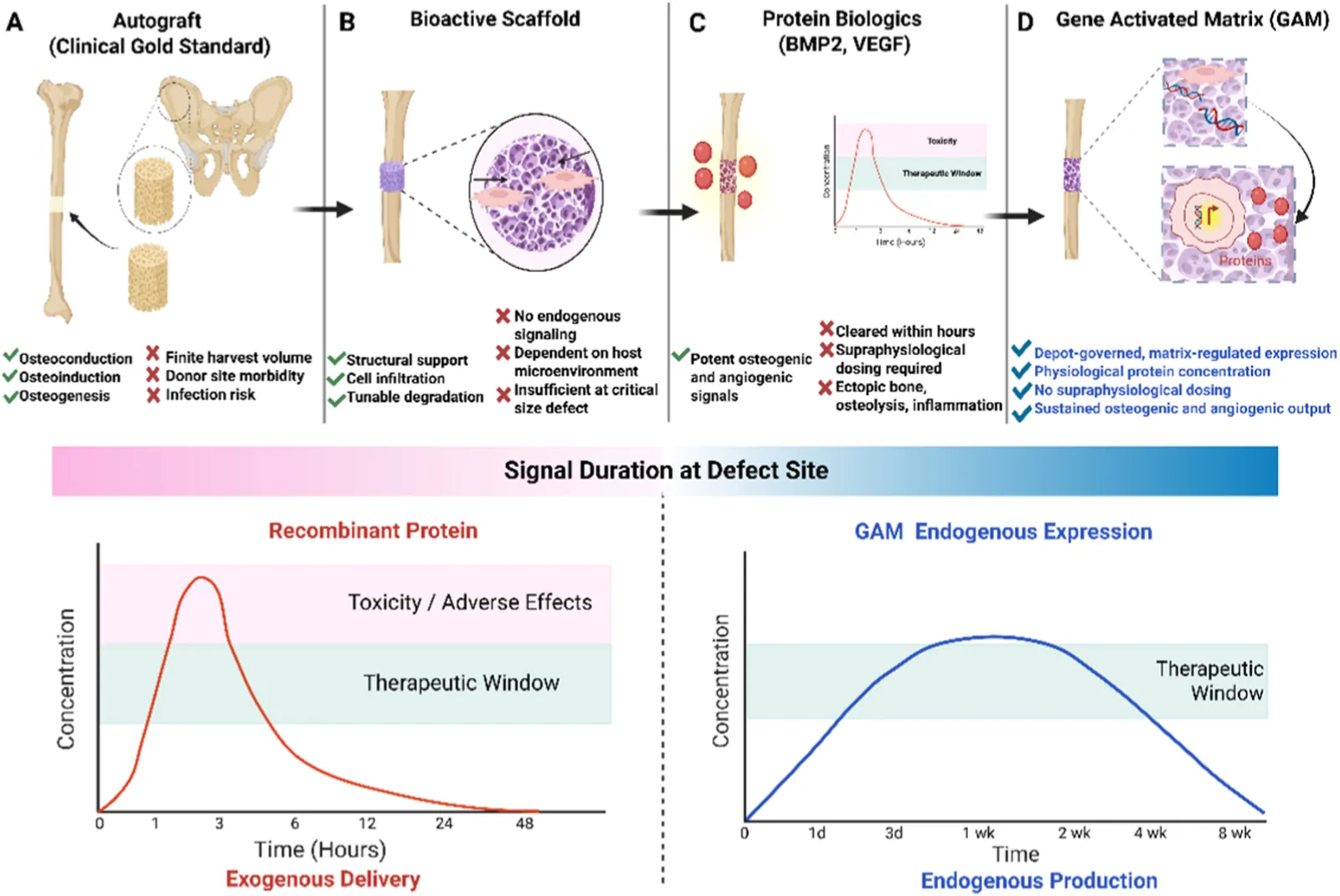
**Comparative overview of bone regeneration strategies and signal duration profiles at the defect site.** (A) Autograft serves as the clinical gold standard but is constrained by finite harvest volume and donor site morbidity. (B) Bioactive scaffolds support cell infiltration but lack intrinsic osteogenic signaling at critical size. (C) Protein biologics are potent but transient, with supraphysiological dosing driving toxicity and ectopic bone formation. (D) GAMs redirect host cells toward sustained endogenous protein production. Bottom panels contrast the pharmacokinetic profiles of exogenous delivery versus GAM-mediated expression, illustrating the therapeutic window advantage of depot-governed, cell-mediated output over days to weeks. (For interpretation of the references to colour in this figure legend, the reader is referred to the Web version of this article.)

Potency alone doesn't solve this problem. Protein biologics can deliver strong osteogenic and angiogenic stimulation, but their presence at the defect site is too transient to sustain repair through to completion [8], [9]. Dose escalation compensates only temporarily, exposing surrounding tissue to supraphysiological concentrations and increasing the incidence of adverse effects. The unmet requirement is duration of signaling, not magnitude of dose. This pharmacokinetic limitation is what redirected the field toward nucleic acids. Instead of delivering proteins itself, the strategy delivers the gene that encodes or regulates it, turning cells already resident at the defect into an ongoing endogenous source of the therapeutic signal.

Nanomedicine provides the delivery framework for that transition. Nucleic acid cargo must resist degradation, achieve cellular internalization, and undergo correct intracellular trafficking before exerting any biological effect. Bone regeneration imposes an additional requirement beyond these three, retention within a spatially defined defect. Viral vectors, lipid nanoparticles, polymeric polyplexes, and calcium phosphate nanoparticles satisfy these requirements through distinct mechanisms and differ substantially in transduction efficiency, expression duration, immunogenicity, and bone-site retention. This performance cannot be considered independently of the material in which the vector is embedded. Scaffold surface chemistry, nano- and microscale topography, porosity, mechanics, and degradation rate govern protein adsorption, cell infiltration, osteogenic signalling, and vector availability to infiltrating cells [10], [11], [12]. The scaffold therefore functions not as an inert carrier but as an active component of the delivery system.

Gene-activated matrices (GAM) integrate both functions within a single implantable platform. Rather than relying on repeated or transient administration of exogenous protein, a GAM retains nucleic acid cargo, either directly or through a delivery vector, within a biomaterial scaffold. Infiltrating host cells internalize this cargo to produce or regulate the therapeutic signal locally [13]. Scaffold composition, architecture, and degradation kinetics determine vector retention duration, the cell populations exposed to the vectors, and whether the 3D microenvironment supports productive transfection [8]. A GAM can accordingly be characterized as a nanomedicine platform in which nanoscale delivery chemistry and scaffold-level materials design converge to govern the spatial and temporal expression of the therapeutic gene. The vector-scaffold interface, rather than either component in isolation, is the principal determinant of GAM performance.

This review traces this convergence from scaffold bioactivity to clinical translation. We first examine how scaffold chemistry, architecture, mechanics, degradation, immunomodulation, and angiogenic activity shape the regenerative microenvironment, before addressing the pharmacokinetic limitations of protein biologics that motivate nucleic acid-based strategies. Viral vectors, lipid nanoparticles, polymeric polyplexes, calcium phosphate nanoparticles, and scaffold-integrated delivery systems are then compared on the basis of transduction efficiency, expression duration, immunogenicity, and bone-site retention. We subsequently examine GAM loading and release architecture, substrate-mediated transfection, and cargo selection across plasmid DNA (pDNA), messenger RNA (mRNA), small interfering RNA (siRNA), and micro-RNA (miRNA). Multi-factor delivery, stimuli-responsive systems, and emerging CRISPR-based gene regulation are addressed next. A claim-level patent landscape analysis closes the review, integrated with the manufacturing, regulatory, safety, and human-scale validation barriers that currently separate experimental GAMs from clinical translation.

## 2 Search strategy and scope

This article is a narrative review with a targeted patent search. A narrative approach was selected because GAM studies vary substantially in scaffold, cargo, vector, defect model, and outcome measure, precluding meaningful quantitative pooling. The review was prepared with attention to SANRA principles for narrative reviews [14].

Literature was searched iteratively in PubMed, Web of Science, Scopus, and Google Scholar from January 2026 and updated during revision in September 2026. Registered clinical studies were identified through ClinicalTrials.gov. Core GAM-related terms were combined with bone regeneration, bone tissue engineering, and osteogenesis, then expanded for each subsection with terms for specific cargos, vectors, biomaterials, biological processes, and translational issues.

English-language, peer-reviewed primary studies published from 2015 onward were prioritized. Older studies and selected reviews were retained where they provided mechanistic, safety, or clinical evidence unavailable in more recent literature. Conference abstracts, preprints, and non-peer-reviewed sources were excluded. Articles were assessed in full text and selected for mechanistic relevance. References were managed in Mendeley, duplicate entries were merged, and bibliographic details and DOIs were checked against PubMed or publisher records.

Screening counts and inter-rater agreement were not recorded, and study-level risk of bias was not assessed, so selection and publication bias cannot be excluded. Limitations of the predominantly small-animal evidence base are addressed in Section 8.

The patent search covered USPTO, WIPO Patentscope, Espacenet, Google Patents, and Lens.org. Eligible records were granted patents and published applications with independent claims relating to bone-targeted or scaffold-integrated gene delivery. The earliest filing date served as the reference date for each patent family, and legal status was updated during manuscript preparation. Table 3 is descriptive and does not constitute a freedom-to-operate opinion. Regulatory information was obtained from FDA product documentation, including approved product labeling.

## 3 Bioactivity in bone tissue engineering

A scaffold does more than fill a bone defect. Its surface chemistry, architecture, mechanics, and degradation behavior shape the local regenerative environment. These properties also determine how a GAM functions by regulating protein adsorption, cell infiltration, nucleic acid retention, and cargo exposure. Scaffold design therefore links material bioactivity with the delivery and expression of therapeutic nucleic acids.

### 3.1 Principles of biomaterial-induced bioactivity

At the material interface, surface chemistry determines how the scaffold interacts with proteins and therapeutic nucleic acids. Hydroxyl, carboxyl, amine, and phosphate groups control wettability, surface energy, and ion exchange, thereby influencing protein conformation and integrin-binding domain accessibility [47], [48]. Carboxyl or amine groups can attract albumin, fibrinogen, and fibronectin, whereas bioinert surfaces show minimal binding under comparable conditions [49]. On hydroxyapatite (HA), calcium affinity and microporosity supported a stable albumin layer within 48 h [50]. These interactions also influence nucleic acid adsorption, making surface chemistry a shared determinant of scaffold bioactivity and vector retention in GAMs.

Physical architecture determines how cells interact with the scaffold and how deeply they can access the cargo. Nano- and microscale features direct protein orientation, cell spreading, and osteoblast organization [47], [48]. Microgrooved and micropatterned surfaces drove osteoblast alignment and differentiation [51], [52]. Pore size, porosity, and interconnectivity govern nutrient diffusion, vascular ingrowth, and access to vector-laden regions [47]. Matrix stiffness adds a mechanical signal that shapes cytoskeletal organization and mesenchymal stem cell (MSC) lineage commitment. Elasticity can promote osteogenic differentiation through an integrin β1 dependent mechanism [53], [54]. Substrate mechanics also modulate polyplex uptake and transgene expression, as discussed in Section 6.3. The same scaffold architecture therefore affects both biological response and gene delivery.

Degradation is a biological determinant as well as a structural process. Its rate, mechanism, and by-products must remain synchronized with tissue formation and remodeling [55], [56]. In GAMs, degradation sets the vector release timeline because matrix breakdown exposes retained cargo to infiltrating cells. No single scaffold property determines osteogenic outcome or GAM performance. Surface chemistry, topography, mechanics, and degradation jointly govern the protein adsorption, cellular infiltration, vector retention, and cargo exposure.

### 3.2 The biological triad of bone regeneration: osteoconduction, osteoinduction, and osteogenesis

Scaffold bioactivity integrates osteoconduction, osteoinduction, and osteogenesis. These overlapping processes support cell and vascular invasion, direct progenitor differentiation, and drive bone formation. GAMs must therefore combine a permissive scaffold environment with effective nucleic acid delivery.

Osteoconduction depends on scaffold architecture and material composition. Filament diameter, pore size, and pore geometry regulate cell attachment, vascular ingrowth, and osteogenic differentiation, although optimal pore dimensions vary across materials and cell types [57], [58], [59], [60], [61]. Material composition further shapes osteoconductivity. HA-containing ceramics, carbon nanofiber composites, and MgO-functionalized silk fibroin scaffolds have all enhanced cell attachment, proliferation, osteogenic activity, and bone formation through mechanisms including ion release and surface-mediated mineral nucleation [62], [63], [64]. In GAMs, the same architecture determines how deeply infiltrating cells reach vector-laden regions of the scaffold.

Osteoinduction directs MSC recruitment and osteogenic differentiation through growth factors, ionic dissolution products, cell-based approaches, or material-intrinsic signals [65]. Bone morphogenetic protein (BMP)-2-loaded scaffolds enhanced bone regeneration and ectopic bone formation [66], [67], [68]. Bioactive ceramics and composite scaffolds promote osteoinduction through endogenous BMP-related signaling activation and ion release [69], [70], [71]. GAMs use an additional route by delivering nucleic acids that instruct infiltrating cells to produce osteogenic signals locally. Their performance therefore depends on both the inductive cargo and the scaffold conditions that support cell infiltration and transfection.

Osteogenesis is the cellular output of bone repair, encompassing osteoblast maturation, collagen deposition, and mineralization. It differs from osteoconduction and osteoinduction, which establish structural and signaling conditions for tissue formation. Committed progenitors, vascular supply, and sustained osteogenic signaling are required through remodeling [65], [72]. In GAMs, that signaling must come from local transgene expression.

Seeded MSCs can provide osteoinductive signals in addition to occupying the scaffold. O-phospho-L-serine (OPS)-functionalized collagen/nHA scaffolds seeded with human MSCs enhanced alkaline phosphatase (ALP) activity, osteopontin (OPN) secretion, and calcium deposition *in vivo* [73]. A decellularized MSC-derived extracellular matrix (ECM) coating on 3D-printed PCL scaffolds improved cell attachment, mineralization, and osteogenic gene expression [74]. In a rat femoral defect, an nHA-PLGA scaffold released vascular endothelial growth factor (VEGF) and fibroblast growth factor-2 (FGF-2) early, followed by sustained BMP-2 delivery. This sequence improved vascularization and osteogenesis compared with simultaneous co-delivery [75]. This temporal logic is also relevant to GAMs, where cargo design and scaffold degradation must support the sequence of repair.

Microfluidic PLGA carriers enabled sequential delivery of VEGF and BMP-2 [76]. In a HUVEC-laden alginate-RGD hydrogel containing MSCs, this design promoted endothelial tunneling and ectopic bone formation. The study illustrates how spatially, and temporally coordinated delivery can support both angiogenesis and osteogenesis, a principle relevant to multi-factor GAM design.

### 3.3 From protein adsorption to osteogenic signaling

Following implantation, scaffold surfaces rapidly acquire a dynamic protein layer through a competitive, time- and concentration-dependent adsorption sequence known as the Vroman effect [77], [78]. Albumin adsorbs first, followed by globulin, fibrinogen, fibronectin, and other plasma proteins. Surface wettability influences the extent of protein exchange. Hydrophobic surfaces favor fibrinogen retention, whereas hydrophilic surfaces permit continued exchange [77].

Fibronectin (FN) and vitronectin (VN) are important mediators of osteogenesis because their activity depends on conformation and integrin accessibility rather than protein abundance alone [79], [80], [81], [82], [83], [84]. FN carries both the RGD integrin-binding motif and the synergistic PHSRN (Pro-His-Ser-Arg-Asn) site, while VN presents RGD alone [79]. Hydrophilic surfaces expose both FN motifs, favouring α5β1 engagement and osteogenic signaling [80], [81]. Co-adsorption of FN and VN on poly(ethyl acrylate) formed a fibrillar network that boosted integrin signaling, BMP-2 retention, and matrix mineralization [84]. In GAMs, the adsorbed protein layer may therefore influence both cell signaling and cellular uptake of nucleic acid cargo (Section 6.3).

Integrins convert adsorbed-layer information into an osteogenic transcription program. α5β1 activates FAK and ERK1/2, driving runt-related transcription factor 2 (Runx2) expression in hMSCs, while β1 silencing abolishes this response [54], [85], [86]. RhoA/ROCK acts in parallel by transmitting cytoskeletal tension, promoting YAP/TAZ nuclear entry and Runx2 amplification [85], [87], [88]. αvβ3 binds a broader range of RGD-containing proteins and associates with BMP receptors, linking integrin signaling with BMP/Smad and Wnt/β-catenin pathways [65], [72], [89]. Downstream of Runx2, Osterix drives the terminal osteogenic gene program, including collagen type I alpha 1 (*COL1A1*), osteocalcin (OCN), and bone sialoprotein, and links matrix formation to angiogenesis through VEGF regulation [1], [90]. These pathways may influence how infiltrating cells respond to therapeutic factors expressed from GAMs.

### 3.4 Classes of bioactive materials

Material class determines how the osteogenic cascade in Section 3.3 is initiated. Bioceramics act through ionic dissolution, natural polymers through receptor recognition, and composites through phase interaction. For GAMs, material class also determines how nucleic acid cargo is bound, retained, and released.

#### 3.4.1 Bioceramics

Bioceramics act through ionic exchange with the surrounding physiological environment. Released Ca^2+^ and PO_4_^3−^ from CaP activate ALP, OPN, and Runx2 through calcium channels and phosphate transporters [91], [92]. Release ratios of 1.5–2 favor osteoinductive behavior, while micropores of 2–10 μm amplify ion exchange and protein adsorption [91], [92]. CaP surfaces form a carbonated apatite layer, sequestering osteoinductive proteins [91], [93]. GAMs use the same mineral affinity to bind nucleic acid vectors [93], [94], although excessive binding can limit vector availability (Section 6.2).

Bioactive glass extends this mechanism to a broader set of therapeutic ions. Silicon stimulates VEGF-coupled osteogenesis, and Li^+^ activates Wnt/β-catenin, upregulating *RUNX2*, ALP, OCN, and OPN in human bone marrow stromal cells (BMSCs) [95]. Zn^2+^ acts as an ALP cofactor that drives collagen deposition and ECM calcification [96], [97]. Co^2+^ stabilizes hypoxia-inducible factor 1-alpha (HIF-1α), enhancing VEGF signaling [95]. Molybdenum-doped mesoporous bioactive glass nanoparticles enhanced BMP-2 production but suppressed VEGF and endothelial tube formation, so ion identity alone can uncouple osteogenesis from angiogenesis [98].

#### 3.4.2 Natural polymers

Natural polymers such as collagen, gelatin, hyaluronic acid, chitosan, and silk fibroin combine biocompatibility with cell-recognition motifs [19], [99], [100], [101]. Collagen type I presents two active motifs, GFOGER and GVMGFO, which engage integrin α2β1 and discoidin domain receptor 2 (DDR2), respectively, activating ERK/MAPK signaling and Runx2 [100]. In 3D hydrogels, integrin α2 additionally recruits p38 MAPK, amplifying osterix expression and mineralization beyond integrin α1 signaling alone [101]. CD44 engagement by hyaluronic acid drives YAP/TAZ nuclear translocation and Runx2 upregulation, independent of substrate stiffness [102]. Collagen-based matrices are frequent GAM carriers, as their hydrophilicity and crosslinkable architecture support soak-loading and sustained vector retention [19], [99].

#### 3.4.3 Synthetic polymers

Synthetic polymers include degradable poly(lactic acid) (PLA), poly(lactic-co-glycolic acid) (PLGA), and polycaprolactone (PCL), and non-degradable polyetheretherketone (PEEK) and polyetherketoneketone (PEKK) [103], [104], [105]. Degradation rate is the defining design variable, tunable across a range no natural polymer can span [12], [104]. PLGA degrades over weeks to months depending on its lactide:glycolide ratio, and its acidic by-products can lower local pH and impair cellular integration if breakdown outpaces tissue remodeling [12], [104]. PCL degrades over 2 to 3 years *in vivo*, among the slowest of the biodegradable polyesters [103], [104]. PEEK and PEKK match cortical bone stiffness with radiolucency and chemical stability. A patient-specific gyroid PEEK/β-TCP scaffold achieved load-bearing bone regeneration clinically [105], [106].

Bioinertness is the trade-off for synthetic polymers' tunable degradation. Hydrophobic, chemically inert surfaces lack integrin-binding sequences and protein-nucleating groups, suppressing FN and VN presentation [103], [107]. This attenuates FAK/ERK, RhoA/ROCK, and Runx2 signaling [51], [108]. PEEK and PEKK tend toward fibrous encapsulation rather than direct bone apposition [105], [109].

Plasma treatment, sulfonation, or coating with HA, collagen, or polydopamine restores integrin-accessible protein conformation [103], [107], [110]. Sulfonated, apatite-coated PEKK induced ectopic bone formation through adenylate cyclase 9-mediated cAMP/PKA signaling [105]. Micropore engineering with Si_3_N_4_/Ta particles restored osteogenic activity without exogenous biologics [109]. In GAM design, synthetic polymers serve as the load-bearing backbone of composite scaffolds, where their degradation rate governs vector release (Section 3.1).

#### 3.4.4 Composite and hybrid materials

Each material class carries a trade-off, namely brittleness for bioceramics, low mechanical strength for natural polymers, and Bioinertness for synthetic polymers. Composite design combines two or more phases to offset these limitations. In GAMs, pairing a nucleic acid-adsorbing mineral phase with a degradable polymer carrier retains vector cargo while providing the osteoconductive environment for infiltrating cells [94], [111].

Pairing a synthetic polymer with a CaP or silicate filler is a common composite strategy that restores ion release and surface nucleation to the bioinert backbone. Strontium- and zinc-doped nano-HA in PLGA bulk scaffolds and electrospun mats matched cancellous bone compressive properties [112], [113], and combined antibacterial and osteogenic activity [114]. In PLGA/bioactive glass composites, silicate dissolution activated endogenous BMP-related signaling and stimulated ectopic bone formation under non-osteogenic conditions [69].

Adding a natural polymer restores the integrin-binding motifs that synthetic backbones lack. Collagen type I coating on 3D-printed PCL/HA scaffolds improved integrin-mediated hMSC attachment and osteogenic differentiation over PCL/HA alone [115]. Mineralized collagen/PLGA composites upregulated Runx2, OCN, and COL-1 in rabbit defect models, with early cell adhesion exceeding the synthetic-only control [116].

Defects spanning multiple tissue zones need more than one biological instruction. A triphasic scaffold with a PLGA/fibrin layer, a poly(L-lactide-co-caprolactone)/bioactive glass interfacial membrane, and a 3D-printed calcium silicate bone layer achieved zone-specific BMSC differentiation and regenerated hyaline cartilage and subchondral bone simultaneously in rabbit and minipig models [117]. Spatial phase arrangement, not material identity alone, governs bioactivity in multi-zone defects.

#### 3.4.5 Functionalized and smart materials

Functionalized and smart materials add a targeted biological cue that the base material cannot provide, through peptide conjugation, ion doping, or stimulus-responsive design. Each strategy has a direct GAM analog. GFOGER, a collagen-mimetic peptide conjugated to MPEG-PCL hydrogel and engaged α2β1 and α11β1 integrins on rat MSCs, activating FAK, ERK, and p38 MAPK and improving defect repair in a rat model [118]. The BMP-2 wrist epitope DWIVA (Asp-Trp-Ile-Val-Ala), co-grafted with RGD on titanium, engaged integrins αvβ3 and α5β1 and BMP receptor type I simultaneously, producing new bone in rat calvarial defects without exogenous growth factor [119]. A BMP-2 transgene in a GAM targets the same receptor, but its product is secreted by transfected host cells rather than presented by the material surface.

Ion doping offers a release-based alternative to surface-bound peptides. Continuous Zn^2+^ release from PLGA/β-TCP scaffolds over 16 weeks activated Wnt/β-catenin and p38 MAPK signaling, promoting MSC adhesion and osteogenic differentiation in a rat femoral condyle model [120].

Stimuli-responsive designs deliver signals triggered by an external or physiological cue. A 3D-printed poly(glycerol sebacate)/poly(3,4-ethylenedioxythiophene):poly(styrenesulfonate) triboelectric scaffold converted physiological deformation into local electrical stimulation. This promoted BMSC chondrogenesis and subsequent endochondral ossification in a rabbit calvarial defect model [121]. The proposed mechanisms included calcium signaling, phosphoinositide 3-kinase/protein kinase B (PI3K-Akt) activation, cytoskeletal remodeling, and vascular invasion [121]. This is material-mediated stimulation rather than gene delivery. Its triggering logic, however, matches pH- and enzyme-responsive GAM release (Section 6). In both, the signal follows a physiological cue rather than a fixed schedule.

### 3.5 Immunomodulatory and angiogenic bioactivity

The immune response is an active determinant of bone regeneration outcome. Neutrophils and macrophages initiate a cascade upon scaffold placement that governs whether healing progresses toward tissue formation or fibrous encapsulation. Physicochemical scaffold properties, including surface topography, wettability, and stiffness, directly regulate this cascade by shaping macrophage polarization [122]. The M1 phenotype drives early inflammation through tumour necrosis factor (TNF-α), interleukin (IL)-1β, and IL-6; the pro-regenerative M2 phenotype resolves it through IL-10. Successful repair requires a controlled M1-to-M2 transition that determines the osteogenic and angiogenic potential of the local microenvironment.

Scaffold design can directly engineer this transition. Nano-scale HA particles induced M2 polarization via the STAT3/c-Maf axis, driving IL-10 secretion that promoted MSC osteogenesis and endothelial tube formation *in vivo* [123]. Submicron calcium phosphate surface topography skewed macrophages toward an M2-like phenotype, with conditioned media enhancing angiogenic tube formation over micron-scale surfaces [124]. Honeycomb titanium nanostructures activated anti-inflammatory macrophage morphologies through cytoskeletal remodeling alone, without exogenous biochemical stimulus [125]. Submicron-surface-treated CaP ceramics additionally enriched macrophage-derived exosomes with miR-142a-5p, suppressing PTEN and activating AKT in recipient MSCs to drive osteogenic differentiation [126].

The coupling between immunomodulation and angiogenesis is mechanistically direct. CD301b^+^ macrophage subsets mediate bioceramic-induced vascularization through the calcineurin/NFATc1/VEGF axis; their depletion abolishes angiogenesis entirely [127]. Mg^2+^-releasing hydrogels activate both macrophages and T lymphocytes, establishing an anti-inflammatory milieu that elevated VEGF expression, enhanced endothelial tube formation, and accelerated bone repair *in vivo* [128]. Immunomodulation and angiogenesis are closely interconnected processes, as scaffold surface properties activate both simultaneously, and neither proceeds independently at the defect site.

Scaffold surface properties can initiate this transition, but sustaining it across the full repair timeline requires additional biological intervention. GAMs represent one such strategy. Single-cell transcriptomic profiling of femoral defect hematomas showed that cell-based GAMs generated M2-skewed macrophage populations with IL-10, CD163, and PPARG expression, while recombinant protein controls showed net pro-inflammatory cytokine dominance [129]. Immunomodulation is therefore a first-class GAM design parameter, not a secondary scaffold property.

## 4 Biologics in bone regeneration

Critical-size bone defect repair requires more than a structurally competent scaffold. Restoring the osteogenic and angiogenic environment disrupted by injury demands exogenous bioactive signals. Protein-based biologics, namely growth factors, autologous concentrates, and bioactive peptides, represent the most clinically established means of supplying them. Short half-life, dose sensitivity, and uncontrolled release are intrinsic liabilities. None has been resolved through formulation refinement alone.

### 4.1 Growth factors: BMPs, VEGF, and FGF-2

BMP-2 and BMP-7 are potent osteoinductive agents whose clinical application is constrained by a pharmacokinetic liability that potency alone cannot resolve. The BMP-2/7 heterodimer achieves fracture healing at doses 20-fold lower than BMP-2 homodimer in a rat femoral defect model [130]. This potency advantage arises from a distinct receptor mechanism, with activin receptor type-1 exclusively phosphorylating Smad1/5/8 and BMP receptor type-1 performing a kinase-independent scaffolding function within the heterodimer complex [131]. Recombinant BMP-2 is approved for spinal fusion and long bone repair, with improved fusion rates and reduced reoperation over autograft confirmed across multiple trials [132]. The therapeutic window is narrow. Ectopic bone formation, osteolysis, inflammatory soft tissue reactions, and PPARγ-mediated adipogenesis all arise from supraphysiological dosing, and complication risk rises with dose without proportional gain in outcome [133].

VEGF is the primary molecular driver of vascular invasion in bone repair, signaling through VEGFR2 to drive endothelial proliferation and nutrient delivery to regenerating tissue [2], [134]. Its expression peaks in the fracture hematoma during the early inflammatory phase, coordinating vascular invasion across both endochondral and intramembranous ossification [134]. The dose-response relationship is narrow. Optimal concentrations induce a pro-osteogenic endothelial phenotype via Notch1 signaling, while excess VEGF increases osteoclast recruitment and suppresses Runx2 and OCN, producing net impairment of bone formation [135].

FGF-2 contributes to bone regeneration as a mitogenic and proangiogenic regulator rather than a direct osteoinductive agent. Signaling through FGFR1–4 via MAPK/ERK and PI3K/AKT pathways, it expands the osteoprogenitor pool, enhances VEGF expression, and supports early vascularization [136]. The dose-response is stage-dependent. Transient low-dose exposure promotes osteogenic differentiation; sustained or high-dose application maintains cells in an undifferentiated state and inhibits mineralization [136]. Clinical trials in periodontal and craniofacial defects confirm dose-dependent improvements in bone fill, with effect magnitude contingent on delivery timing and concentration [136].

### 4.2 Platelet-rich plasma and endogenous growth factor concentrates

Platelet concentrates provide a clinically accessible means of delivering multiple endogenous growth factors to the repair site without recombinant proteins or gene transfer. Platelet-rich plasma (PRP), platelet-rich fibrin (PRF), and plasma rich in growth factors (PRGF) are each prepared by differential centrifugation of whole blood, yielding supraphysiological platelet loads within a fibrin matrix [137], [138]. The retained payload, namely platelet-derived growth factor (PDGF), transforming growth factor (TGF-β1), VEGF, and insulin-like growth factor (IGF-1), is released locally over time. Centrifugation speed and duration are primary sources variability, generating measurably distinct release profiles across subtypes [139].

PRGF and PRF differ in their biological effects, each producing distinct payload profiles with measurable functional consequences. PRGF exposure promoted osteoblast differentiation, as evidenced by increased ALP activity and upregulation of the osteogenic markers RUNX2 and SP7 *in vitro* [140]. Direct contact with the PRGF clot further amplified ALP activity, suggesting that the fibrin matrix contributes actively to cell behavior rather than simply serving as a passive carrier [140]. PRF matrices provided sustained release of TGF-β1 and PDGF which supports extracellular matrix production by osteoblasts [141]. Despite these promising biological effects, clinical outcomes have remained inconsistent [142], [143].

This inconsistency is partly due to differences in preparation protocols, which generate fibrin matrices with varying growth factor content and release profiles [144]. As a result, platelet concentrates often show batch-to-batch variability and limited control over growth factor delivery.

### 4.3 BMP-derived peptides and integrin-binding sequences

Efforts to replicate growth factor signaling while avoiding the limitations of recombinant proteins have driven the development of bioactive peptide therapeutics. Osteogenic signaling initiates at discrete epitopes rather than across the whole protein surface [145]. Short synthetic peptides spanning those regions engage the same receptor machinery at a fraction of the molecular weight. Synthesis reproducibility is greater and off-target risk is reduced, though activity depends critically on spatial presentation and local concentration.

BMP-2 contains several peptide regions that retain osteogenic activity. One such sequence, KIPKASSVPTELSAISTLYL elevated ALP activity, upregulate OCN expression, and promoted ectopic bone mineralization *in vivo* [146]. Another peptide DWIVA, activated p38 signaling independently of a canonical Smad pathway and induced osteogenic differentiation without additional soluble induction factors [145]. When combined with cyclic RGD on titanium surfaces, DWIVA further enhanced the expression of osteogenic markers including *Runx2*, *Col1A1*, and Osterix and promoted new bone formation in rat calvarial defects [119].

Integrin-binding peptides promote osteogenic signaling through cell-adhesion signaling rather than direct activation of BMP receptors. Controlled co-presentation of RGD with the FN synergy motif PHSRN enhanced α5β1 engagement, upregulated Runx2 in hMSCs, and produced bone formation in rat calvarial defects without the need for exogenous growth factor supplementation [147]. Bifunctional surfaces combining both RGD with the BMP-2 derived peptide produced stronger osteogenic commitment in hBMSCs than either peptide alone [148]. Despite these promising effects, the activity of both BMP-derived and integrin-binding peptides remains highly dependent on their local concentration and spatial presentation, motivating the development of nucleic acid-based strategies discussed in Section 4.4.

### 4.4 Shared limitations and the rationale for nucleic acid strategies

A shared pharmacokinetic constraint underlies the limitations of all biologic classes reviewed above [8], [133]. Fibrin architecture governs an uncontrolled platelet concentrate burst, and peptide activity collapses without precise spatial presentation. The comparative expression kinetics of protein delivery versus GAM-mediated endogenous production are shown in Fig. 1.

The clinical response to this constraint has been dose escalation. BMP-2 clearance before adequate receptor engagement forces supraphysiological dosing, with ectopic ossification, osteolysis, systemic inflammation, and prohibitive cost all documented consequences [133]. Autologous concentrates avoid exogenous protein entirely, yet their uncontrolled, batch-dependent release kinetics produce small and inconsistent clinical effects across meta-analyses [142], [143]. The limitation is structural, not a matter of formulation.

Nucleic acid strategies address this problem at the gene level. Rather than delivering a protein that degrades, they instruct target cells to produce that protein endogenously, at physiologically regulated concentrations, for a duration determined by construct design. BMP-2 complementary DNA delivery in rat segmental defects achieved complete healing at doses 2-3 orders of magnitude below the recombinant protein dose required for the equivalent outcome [8]. Endogenous synthesis additionally yields natively folded, post-translationally modified protein, free from the conformational alterations that reduce activity and provoke immune responses in recombinant preparations [8], [9]. Realizing this advantage requires a delivery vehicle capable of protecting nucleic acid cargo from degradation, facilitating cellular uptake, and sustaining expression across the phases of repair. These are constraints specific to nucleic acid delivery, since recombinant protein delivery requires no comparable protection or intracellular targeting, and their resolution is the central engineering problem of Section 5.

## 5 Delivery systems for nucleic acids in bone tissue engineering

Delivery architecture shapes transgene expression as decisively as nucleic acid cargo. Viral, lipid-based, polymeric, calcium phosphate, and scaffold-integrated platforms each present a distinct profile of transduction efficiency, expression duration, immunogenicity, and bone-site retention, as summarized in Fig. 2.

**Fig. 2:**
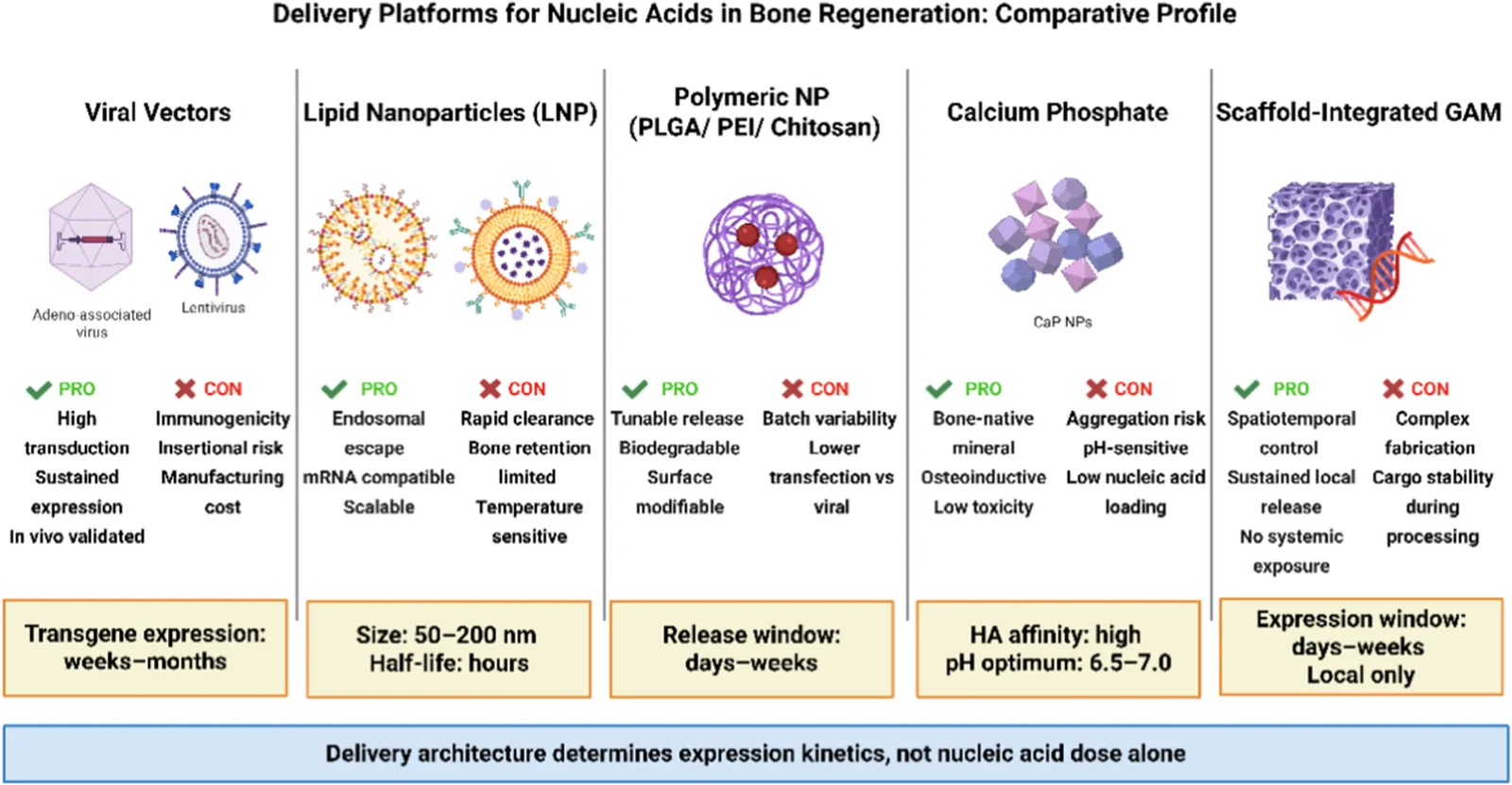
**Comparative profiles of five nucleic acid delivery platforms for bone regeneration.** Platforms are compared across transduction efficiency, expression duration, immunogenicity, and retention at the bone defect site. Viral vectors provide efficient transduction and potentially sustained expression, but all may provoke vector- or transgene-directed immunity, whereas insertional mutagenesis is principally associated with integrating vectors such as lentivirus. Lipid nanoparticles enable endosomal escape and mRNA compatibility but lack mineral affinity and clear rapidly from the bone-poor vasculature. Polymeric nanoparticles offer tunable biodegradable release with lower transfection efficiency. Calcium phosphate nanoparticles are bone-mimetic and osteoinductive but limited by aggregation and nucleic acid loading. Scaffold-integrated GAMs provide spatiotemporal control and sustained local expression without systemic exposure at the cost of more complex fabrication.

### 5.1 Viral vectors

Viral vectors exploit host cell endocytic machinery for high-efficiency intracellular delivery of nucleic acid cargo. Adenoviral, adeno-associated (AAV), and lentiviral vectors differ systematically in genome fate, expression duration, transduction efficiency, and immunogenicity, properties that map directly onto the biological requirements of bone repair (Table 1).

**Table 1:** Comparative properties of adenoviral, AAV, and lentiviral vectors for bone gene therapy.

Property	Adenoviral	AAV	Lentiviral	References
Genome fate	Episomal dsDNA, non-integrating	Episomal concatemer, non-integrating	Stable genomic integration	[15], [16], [17]
Expression duration	Several weeks	Months to years	At least 8 weeks *in vitro*, sustained	[15], [17]
Transduction efficiency	High in dividing and non-dividing cells	<9–20% in human MSCs *ex vivo*; high *in vivo* bone tropism (serotype-dependent)	Moderate/High	[15], [16], [18]
Immunogenicity	High	Serotype-dependent; pre-existing neutralizing antibodies restrict redosing	Low/Moderate	[15]
Principal limitation	Immunogenicity restricts repeat dosing	Serotype-dependent immunity; limited MSC transduction *ex vivo*	Vector-design-dependent genotoxicity risk	[15], [16], [18]

First-generation adenoviral vectors are replication-deficient viruses that enter cells through receptor-mediated uptake and deliver non-integrating double-stranded DNA (dsDNA) to the nucleus, producing transgene expression within 24–48 h [15]. This rapid, transient profile suits early fracture-repair induction but not the sustained signaling large defects require. Innate and adaptive immunity develop rapidly after *in vivo* administration, and pre-existing anti-adenoviral immunity, common in adults, further limits repeated dosing [15]. These properties confine adenoviral gene therapy in bone largely to *ex vivo* protocols and proof-of-concept preclinical studies.

In a rat calvarial defect model, adenovirus-transduced bone marrow stromal cells expressing BMP-2 were seeded on a poly(L-lactide-co-ε-caprolactone) scaffold, achieving 43.4% defect closure after 8 weeks. This outperformed combined BMP-2 and VEGF-A delivery at a 1:4 ratio [149]. A second comparison found *ex vivo* MSC transduction outperformed direct *in vivo* delivery, with *in vivo* BMP-2 gene transfer producing 28% new bone volume fraction at 56 days [150]. Neither study examined immune response at the graft site.

Adeno-associated virus (AAV) depends strongly on serotype selection. Among 18 natural and engineered variants tested in a murine fracture model, AAV8, AAV9, and AAV-DJ achieved the highest transduction efficiency at the fracture site [16].

AAV is less inflammatory than first-generation adenovirus, but it is not immunologically inert. Vector genomes can activate innate sensing pathways, including TLR9-dependent signaling, and capsid or transgene antigens can elicit neutralizing antibodies and cytotoxic T-cell responses that eliminate transduced cells [151]. Antibodies generated after the first administration can also impede redosing [151]. A multicountry seroprevalence analysis of 502 adults and 50 children found neutralizing antibodies against clinically relevant AAV serotypes in a substantial proportion of participants, highest for AAV1, lowest for AAV5, and increasing with age [152]. These immune barriers operate independently of the transduction and biodistribution profile a given serotype otherwise offers.

Biodistribution follows a similar serotype dependence. Under a constitutive promoter, systemic AAV8 delivery produced high-level expression not only in bone but also in liver, heart, spleen, and kidney [16]. Replacing the constitutive promoter with the osteoblast-lineage promoters *Sp7* or the 2.3-kb Col1a1 fragment (Col2.3) restricted expression toward osseus tissue, with *Sp7* nearly eliminating liver expression and Col2.3 achieving a smaller reduction [16]. Promoter restrictions limits where the transgene is expressed, but it does not necessarily prevent vector genomes from reaching non-target organs, a distinction the cited study did not resolve at the DNA level.

Despite this *in vivo* promise, AAV fails critically in the *ex vivo* MSC platform underlying most regional bone gene therapy strategies. AAV2 and AAV6 reached only 3.6% and 8.8% transduction efficiency in human bone marrow-derived MSCs, and 6.2% and 20.2% in adipose-derived MSCs, even at high multiplicities of infection. BMP-2 production was insufficient to drive osteogenic differentiation *in vitro* [18]. Under the same conditions, a lentiviral two-step transcriptional amplification system (TSTA) produced abundant BMP-2 secretion and robust mineralization [18]. Bone targeting efficiency *in vivo* does not predict gene delivery success in *ex vivo* human MSCs.

A head-to-head lentiviral TSTA-BMP-2 comparison found transduction of 90.2% in adipose-derived MSCs and 80.4% in bone marrow-derived MSCs, showing cell source shapes vector performance and donor-site selection [17]. Lentiviral BMP-2-transduced human MSCs encapsulated in a gelatin hydrogel and implanted intramuscularly in immunodeficient mice, produced vascularized bone with trabecular architecture within 14 days, with GFP tracking confirming implanted-cell participation [153]. Sustained lentiviral expression can substitute for exogenous growth factor in engineered cell-scaffold systems, though 14-day intramuscular models do not capture the mechanical and immunological demands of load-bearing skeletal repair.

Genomic integration, the property that sustains lentiviral expression, also raises insertional mutagenesis concerns. Self-inactivating (SIN) third-generation vectors reduce this risk by deleting the promoter-enhancer activity of the long terminal repeats, and animal studies have not detected preferential integration near cancer-related genes [15]. Self-inactivation, however, does not eliminate risk. In a clinical study of elivaldogene autotemcel, a SIN-lentiviral hematological malignancy developed in 7 of 67 treated patients [154]. Predominant malignant clones carried integrations involving *MECOM-EVI1* or *PRDM16* together with additional somatic mutations, and the vector's internal promoter-enhancer was identified as one potential contributor among several, alongside transgene overexpression and conditioning regimen [154]. Integration near *MECOM* was common even in patients who did not develop cancer, indicating that persistent clonal expansion and additional genetic abnormalities, not proximity to an oncogene alone, distinguished the malignant cases [154]. A broader synthesis across hematopoietic and immune-cell gene therapy shows SIN design lowers but does not remove genotoxic risk, with vector-copy number, internal promoter choice, and underlying disease among the contributing factors [155]. These findings originate in hematopoietic stem-cell therapy, not bone GAM, and do not establish an incidence of malignancy for regional lentiviral BMP-2 delivery.

The limitation of viral vectors in bone gene therapy is not a lack of preclinical evidence but an unresolved design conflict. Adenoviral systems match the early induction window but lose efficacy as adaptive immunity develops after first administration. AAV avoids this constraint but fails to transduce human MSCs at therapeutic levels in the *ex vivo* platforms clinical protocols required. Lentiviral integration sustains expression but introduces mutagenesis risk that no regulatory dataset has resolved. These trade-offs, not evidentiary gaps, define the design space non-viral and scaffold-integrated strategies must occupy.

### 5.2 Non-viral vectors

Non-viral delivery systems trade immunogenicity and integration risk for lower transfection efficiency and shorter expression duration. Whether this trade-off is acceptable depends on cell populations available for transfection, the required expression window, and tolerance for repeated dosing [8], [9]. Lipid nanoparticles (LNP), cationic polymer polyplexes, and CaP nanoparticles each address this design space through a distinct mechanistic profile, and their translational constraints differ accordingly.

#### 5.2.1 Lipid nanoparticles

Clinical validation in siRNA therapeutics and mRNA vaccines gave lipid nanoparticles a mechanistic and regulatory head start over other non-viral platforms, but skeletal repair imposes a barrier those applications did not face. The four-component architecture of LNPs, comprising an ionizable lipid, helper phospholipid, cholesterol, and PEG-lipid conjugate, facilitates efficient intracellular delivery by promoting pH-dependent endosomal escape through membrane destabilization [156]. Bone is poorly accessible. Low vascular perfusion and a restrictive blood-bone marrow interface limit systemic LNP-mediated delivery. LNPs also lack intrinsic mineral affinity, so particles do not accumulate at the defect site, making local or scaffold-integrated delivery a practical necessity rather than a design preference [156].

Unlike pDNA, mRNA is translated directly in the cytoplasm and does not require nuclear entry, giving it a kinetic advantage that lipid-based formulations can exploit. An LNP formulation containing C12-EPE, DOPE, cholesterol, and DMPE-PEG2k delivered chemically modified mRNA (cmRNA) encoding BMP-2 to rat MSCs, sustaining protein secretion for over 7 days. Expression of the osteogenic markers *Runx2*, Osterix, ALP, collagen I, *OPN*, and *OCN* increased at days 7–14 [21]. *In vivo*, cmRNA-loaded fibrin constructs in rat femoral defects produced measurable bone healing within 2 weeks, at doses far below those required for recombinant protein [21]. Chemical modification of the mRNA, replacing 25% of uridine and cytidine with 2-thiouridine and 5-methylcytidine, reduced detection by TLR7/8 and TLR3 while preserving ribosomal translational fidelity. The fibrin delivery matrix itself, however, was not optimized for mRNA protection or controlled release [21].

Bisphosphonate moieties address the mineral-affinity deficit of conventional LNPs through high-affinity chelation of HA calcium ions. Screening a library of 21 bisphosphonate-lipid materials identified 490BP-C14, which produced 2.5-fold higher protein expression than non-bisphosphonate controls *in vitro*. After intravenous administration in mice, 490BP-C14 showed enhanced bone microenvironment localization, with uptake confirmed in B cells, T cells, monocytes, and marrow endothelial cells [156]. Systemic BMP-2 mRNA delivery via this formulation produced measurable bone tissue secretion without local injection. The lipid was designed so that bone-targeting bisphosphonate groups were exposed on the nanoparticle surface, while amide groups and hydrophobic alkyl chains in the remaining structure facilitated endosomal escape after cellular uptake. However, escape kinetics were not directly quantified and the intracellular release mechanism remains uncharacterized [157].

#### 5.2.2 Polyplexes

Polyplexes form when cationic polymers electrostatically complex with nucleic acids, condensing and protecting genetic cargo for cellular delivery. This mechanism makes them attractive for bone tissue engineering, though the same positive surface charge that drives complexation can also cause cytotoxicity. Polyethylenimine (PEI) is the benchmark polycation in bone gene delivery, with transfection efficiency and cytotoxicity that are equally well-characterized [9]. High positive charge promotes cellular uptake and endosomal disruption via the proton sponge effect, in which proton buffering induces endosomal swelling and membrane rupture. However, the same cationic charge can also induce mitochondrial fragmentation and caspase-9-mediated apoptosis in human MSCs, attenuating the osteogenic capacity that efficient transfection is intended to activate [158].

The osteogenic performance of polyplexes depends as much on the delivery strategy as on the vector composition. Intraoperative bioprinting of PEI-pBMP-2 polyplexes into rat calvarial defects achieved a 1.94-fold improvement in bone volume fraction over empty controls at 6 weeks, outperforming ex situ delivery by 1.33-fold [20]. 3D construct geometry amplified this further, with RUNX2 expression 75-fold higher in bioprinted constructs than in 2D transfected cultures, establishing that the defect microenvironment drives osteogenic output independently of vector design [20]. Non-specific uptake by off-target cells remains a safety concern for in situ polyplex delivery. GO-PEG-PEI (graphene oxide–polyethylene glycol–polyethylenimine) nanocomplexes loaded with miR-29b mimic in a thermosensitive chitosan hydrogel produced incomplete defect filling at 2 months in rat skull defects. The endosomal escape mechanism for GO-based carriers was not characterized [26]. Incomplete repair in the GO system reflects a broader constraint of miRNA-only cargo, where post-transcriptional regulation alone is insufficient to drive complete defect bridging without a concurrent osteoinductive signal [26].

High transfection efficiency does not necessarily translate into better bone regeneration. Although PEI effectively delivers genes, it can induce DNA damage, oxidative stress, mitochondrial dysfunction, and apoptosis in hMSCs, impairing their osteogenic potential [158]. To address this, the genome-protective delivery system (GuaRD) co-delivered pRUNX2 with RS-1, a DNA repair activator. While GuaRD and conventional PEI achieved similar transfection efficiencies (∼15–16%), GuaRD reduced cellular damage and enhanced osteogenic marker expression and mineralization in hMSC spheroids [158]. These findings suggest that preserving cell health after transfection may be as important as achieving efficient gene delivery, although validation in *in vivo* bone defect models is still needed.

Polymer architecture influences cytotoxicity as much as charge density. A 64-arm star-PLL polypeptide achieved two-fold higher luciferase expression than PEI and 130-fold higher than linear PLL in collagen-glycosaminoglycan scaffolds. Host-cell transfection was confirmed *in vivo* at 7 days after cell-free subcutaneous implantation in rats [19]. Star-PLL supported MSC metabolic activity without measurable cytotoxicity. This is a profile that PEI cannot match at equivalent transfection doses. However, the *in vivo* osteogenic output ofstar-PLL systems has not been benchmarked against PEI in load-bearing defect models.

#### 5.2.3 Calcium phosphate nanoparticles

Calcium phosphate (CaP) nanoparticles are the only non-viral vector class in which carrier and therapeutic function are inherently aligned. High affinity for the phosphate backbone of nucleic acids enables stable complexation of plasmid DNA (pDNA), siRNA, and miRNA without cationic polymer bridging [9], [93]. Under acidic lysosomal conditions, the particle dissolves and releases its cargo through a physiologically triggered mechanism, avoiding the proton sponge cytotoxicity that constrains PEI-based systems. Carrier osteogenicity and nucleic acid delivery therefore reinforce rather than compete with each other, a design advantage absent from all other non-viral platforms [93].

Protamine coating extends CaP nanoparticle function beyond gene delivery into antibacterial activity, addressing two obstacles in a single carrier design. Multi-shell CaP nanoparticles loaded with pDNA encoding BMP-2 and IGF-1 achieved transfection efficiency comparable to Lipofectamine in MC3T3-E1 cells. A 1:4 BMP-2:IGF-1 ratio produced the highest Runx2 expression and calcium deposition *in vitro* [159]. Collagen scaffolds loaded with these particles confirmed local protein expression at 28 days in rat cranial defects. Protamine additionally inhibited *Streptococcus mutans* and *Actinobacillus actinomycetemcomitans*, a practical advantage in infection-prone defect environments [159]. Slight cytotoxicity was noted relative to mock controls, though it did not differ across CaP formulations, implicating transfection stress rather than carrier-specific toxicity.

Combining osteogenic gene expression with inflammatory silencing in a single CaP construct addresses a design gap that neither biologic delivery nor single-payload vectors can fill. A triple-functionalized system delivered pDNA encoding BMP-7, pDNA encoding VEGF-A, and siRNA targeting TNF-α, each loaded onto separate PEI-coated CaP nanoparticles with a silica shell and combined into an injectable paste [24]. OCN, Runx2, and SP7 expression was highest in the triple group at day 10. Near-complete defect filling was confirmed by histology at day 21, with no infection, severe inflammation, or necrosis observed [24]. This multi-cargo architecture anticipates the integrated design logic of gene-activated matrices. It is particularly relevant to compromised defect environments where inflammation and osteogenesis must be addressed concurrently, though remodeling beyond day 21 was not assessed.

### 5.3 Scaffold-integrated delivery

Free vectors delivered to a defect site are rapidly cleared by interstitial fluid and diluted before sufficient cellular uptake can occur. Scaffold integration retains vectors at the defect, enables sustained release as the matrix degrades, and presents nucleic acid cargo to infiltrating host cells over a defined temporal window [8], [9]. Far from inert, the scaffold material determines vector retention, cell infiltration kinetics, and the microenvironmental context of transfection. As established in Section 3, surface chemistry, porosity, and degradation kinetics govern cell infiltration and microenvironmental context alike. These same material variables set the boundaries of vector retention and release, a constraint inseparable from scaffold material selection.

This functional interdependence was demonstrated in a cell-free system. Star-PLL-pDNA nanomedicines incorporated into collagen-glycosaminoglycan matrices and implanted subcutaneously in rats produced extensive host cell infiltration and confirmed transgene expression at 7 days, without pre-seeded cells [19]. Scaffold composition was decisive, with collagen-glycosaminoglycan matrices outperforming collagen-ceramic composites in transgene expression, confirming that material selection shapes delivery performance as directly as vector design. A cell-based approach transfected BMSCs with cmRNA encoding BMP-2 and VEGF-A before seeding on a collagen scaffold for implantation into rat calvarial defects [160]. The 1:1 BMP-2:VEGF-A group produced the greatest bone fill by micro-CT at 4 and 12 weeks, with histology confirmed more mature mineralized bone than either single-factor group [160]. BMP-2 peaked at 24 h and VEGF-A at 16 h post-transfection, delivering osteogenic and angiogenic signals early and transiently, aligned with the inflammatory-to-repair transition rather than the remodeling phase. This temporal profile is both the strength and the limitation of mRNA-scaffold systems. Sustaining transgene expression across the full repair timeline requires a structurally different delivery architecture, the gene-activated matrix addressed in Section 6.

### 5.4 Nucleic acid cargo considerations

The delivery carrier determines cellular targeting and uptake, whereas the therapeutic outcome is governed by the nucleic acid cargo. Cargo selection is a design decision, not a default, and one that delivery platforms in bone repair have not consistently optimized [9]. Table 2 summarizes how pDNA, mRNA, siRNA, and miRNA differ in mechanism, kinetics, and constraint.

**Table 2:** Comparative properties of nucleic acid cargo formats in bone repair.

Cargo Type	Mechanism	Onset	Duration	Key Advantage	Key Limitation	Ref
pDNA	Nuclear transit, episomal expression	Delayed (nuclear entry required)	Days–weeks	Sustained protein secretion	Inefficient in post-mitotic cells; low integration risk	[19], [20]
mRNA	Direct cytoplasmic translation	Hours	1–2 days peak, then declines	Rapid onset, no nuclear entry needed	Short-lived; requires protective delivery	[21], [22], [23]
siRNA	RISC-mediated mRNA degradation	Rapid, sequence-specific	Short (requires replenishment)	Precise silencing of inflammatory mediators	Short half-life; off-target silencing risk	[9], [24], [25]
miRNA/antagomirs	Post-transcriptional network modulation	Variable	Variable	Broad regulatory reach, no exogenous transgene needed	Off-target unpredictability; insufficient alone for full osteogenic commitment	[9], [26]

Representative findings illustrate these differences in practice. Plasmid DNA drove a 75-fold amplification of RUNX2 in 3D constructs relative to monolayer culture [19], [20]. Co-delivery of TNF-α siRNA and BMP-7 plasmid DNA in a calcium phosphate construct enhanced bone formation beyond either therapy alone [24]. AntagomiR-138 complexed with nano-hydroxyapatite and incorporated into a collagen-HA scaffold reduced miR-138 expression 2.7-fold in human MSCs [35].

No single cargo format spans the full temporal demands of bone repair, and cargo optimization alone cannot resolve these constraints. What converts discrete nucleic acid formats into a coherent repair strategy is a delivery architecture that physically retains, protects, and presents each cargo to infiltrating cells across a matrix-governed release timeline.

No single vector class is optimal across bone repair applications. The appropriate choice depends on clinical context. Viral vectors suit defects where sustained high-level expression justifies the immunogenicity and, for integration vectors, genotoxicity cost, and where repeated dosing is not required [151], [154], [155]. Lipid nanoparticles and polyplexes suit transient, single-administration windows. Polyplex cationic charge enables transfection but also drives the mitochondrial and apoptotic signalling that limits it [158]. LNPs lack intrinsic mineral affinity, so scaffold integration is required to deliver them to the defect site [156]. Calcium phosphate nanoparticles extend toward multi-cargo, staged release, but adsorptive loss of co-loaded cargo remains unresolved [94]. Thus, no single vector platform simultaneously optimizes cargo protection, delivery efficiency, and cytocompatibility, underscoring the importance of integrating vector selection with scaffold design in GAM development.

## 6 Gene-activated matrices: concept, design, and mechanisms

GAMs resolve a limitation none of their component strategies can address independently. Scaffolds without genetic instruction depend on endogenous signaling that fails at critical-size defect volumes. Nucleic acid cargo delivered without structural retention generates expression too brief and too diffuse to drive repair at that scale. Combining scaffold, vector, and cargo into the single construct described earlier resolves both limitations at once. Expression timeline is now governed by matrix degradation rather than vector half-life [8], [13].

### 6.1 From scaffold to signaling source: the GAM concept

The sequential stages of GAM action, from scaffold implantation through cell infiltration, endosomal escape, transgene expression, and bone formation, are illustrated in Fig. 3. The foundational GAM study entrapped pDNA encoding parathyroid hormone within a collagen sponge implanted into canine segmental defects, sustaining retention and transgene expression for at least 6 weeks [13]. Wound repair fibroblasts invading the sponge were the primary transfected population, and bone formation was dose- and time-dependent, establishing that GAM output is inherently regulatable through cargo loading parameters [13]. The mechanism was intrinsic rather than engineered. The plasmid, as a high-molecular-weight polyanion, could not diffuse through the collagen carrier, making matrix retention a property of the system itself rather than a fabrication constraint [13].

**Fig. 3:**
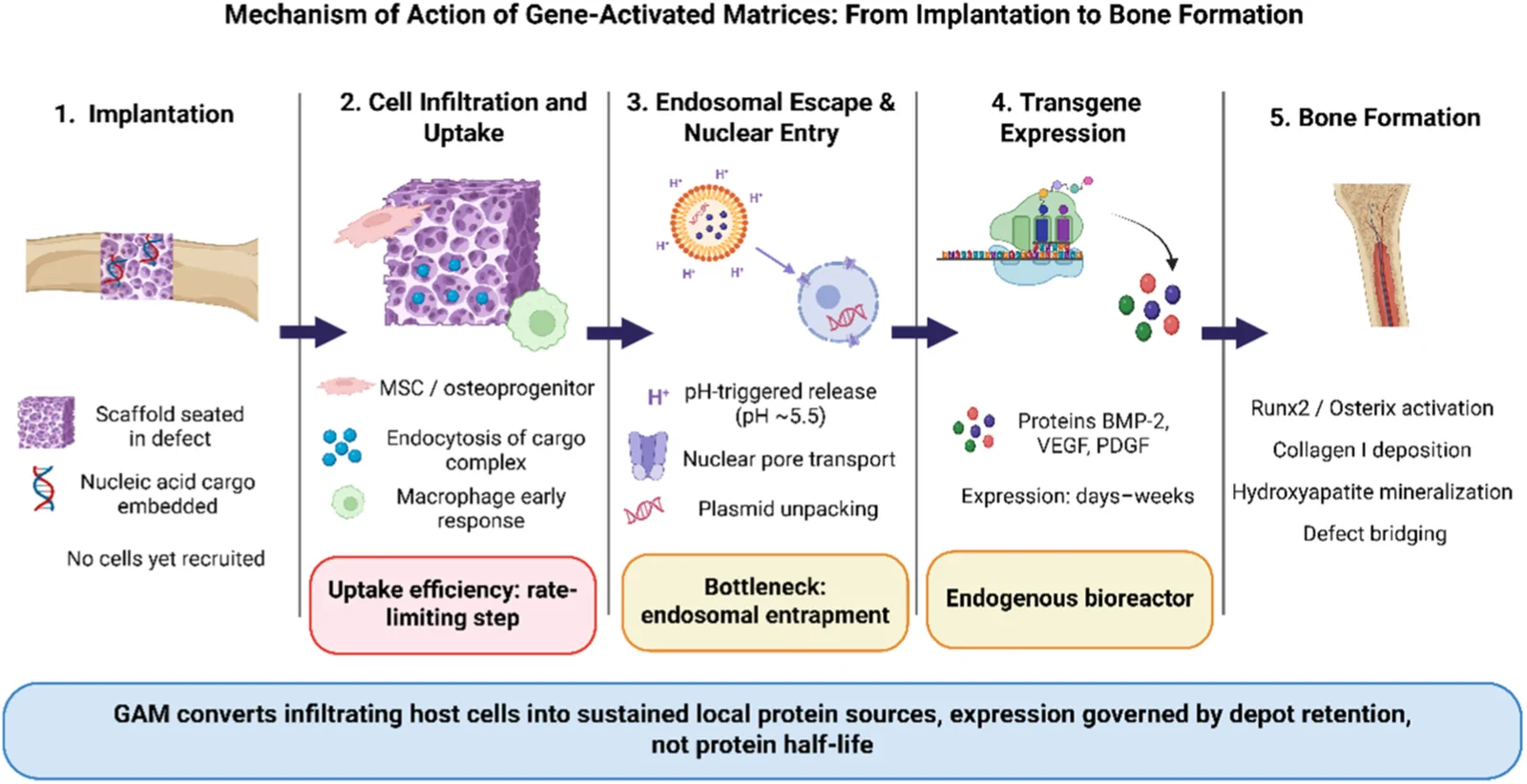
**Mechanism of action of gene-activated matrices: from implantation to bone formation.** At implantation, nucleic acid cargo is embedded within the scaffold matrix with no cells yet recruited to the defect. Infiltrating MSCs, osteoprogenitors, and macrophages internalize the cargo complex through endocytosis, with uptake efficiency representing the rate-limiting step. Endosomal acidification at pH ∼5.5 triggers cargo release, followed by nuclear pore transport and plasmid unpacking. Transfected cells function as an endogenous bioreactor, producing BMP-2, VEGF, and PDGF over days to weeks. Bone formation proceeds through Runx2 and Osterix activation, collagen I deposition, HA mineralization, and defect bridging.

Contemporary GAM design functionalizes the scaffold with gene therapy nanomedicines to provide spatiotemporal control over host-cell transfection as osteoprogenitor populations infiltrate the construct [19]. The scaffold retains nucleic acid cargo at the defect, prolongs nanomedicine availability, and reduces burst release over a clinically relevant window, functions that direct injection cannot replicate [13], [19]. Localized transgene expression produces osteogenic regulators at biologically relevant concentrations, governed by depot retention rather than protein half-life [9], [161]. The construct still depends on an adequate local responding cell population to produce this effect [15].

### 6.2 Nucleic acid loading strategies and release architecture

Three loading strategies and three release mechanisms govern nucleic acid availability at the defect, as summarized in Fig. 4, and their selection has direct consequences for expression duration and spatial distribution [25]. Release rate is entirely governed by matrix physicochemistry, with no mechanism to modulate cargo availability independently of scaffold degradation [13].

**Fig. 4:**
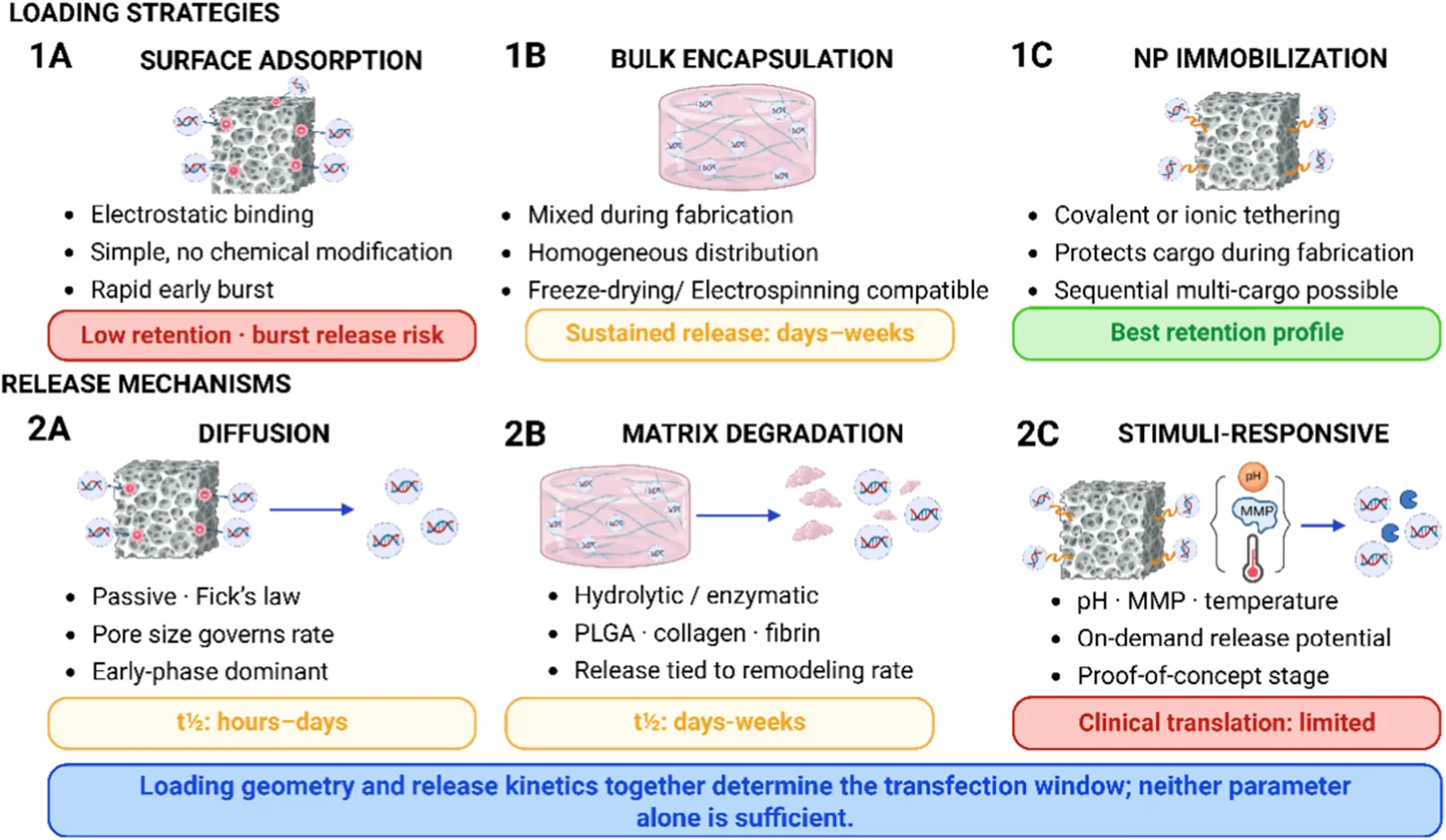
**Nucleic acid loading strategies and release architecture in gene-activated matrices.** Loading by surface adsorption offers simplicity but carries burst release risk and low retention. Bulk encapsulation distributes cargo homogeneously during fabrication, supporting sustained release over days to weeks. Nanoparticle immobilization through covalent or ionic tethering provides the strongest retention profile and enables sequential multi-cargo loading. Release occurs through diffusion governed by Fick's law, matrix degradation coupled to hydrolytic or enzymatic remodeling, or stimuli-responsive mechanisms triggered by pH, matrix metalloproteinases, or temperature. The stimuli responsive route remains at proof-of-concept stage without clinical translation.

Pre-formed nanoplexes soak-loaded into porous scaffolds provide nucleic acid condensation and enzymatic protection without altering scaffold architecture [25]. This extends the transfection window beyond what physical entrapment permits. Chitosan-pDNA nanoplexes at N/P 20 sustained MSC transgene expression for 28 days in collagen-HA scaffolds, with bone formation exceeding gene-free controls by more than 11-fold in a rat calvarial defect [99]. Plasmid sequence identity proved an independent determinant within this architecture; a codon-modified BMP-2-Advanced construct accelerated osteoprogenitor differentiation and defect repair beyond a standard BMP-2 plasmid delivered by the same method [162].

Scaffold macromolecular composition governs depot behavior independently of loading strategy. Mineral affinity for nucleic acid vectors is not a fixed property. Moderate binding supports sustained retention, as described in Section 3. Beyond a certain surface area or binding capacity, the same electrostatic and chemical interactions sequester vector past the threshold needed for cellular uptake [19], [94]. Glycosaminoglycan matrices prolonged star-PLL nanomedicine retention through electrostatic sequestration of cationic complexes, whereas mineral-containing matrices reduced available vector through competing ionic interactions with the ceramic phase [19]. A dual pBMP-2 and pVEGF star-PLL construct nonetheless produced 6-fold greater bone volume than scaffold alone at 4 weeks, as ceramic osteogenic activity offset reduced nanomedicine availability [111]. Scaffold chemistry and transfection output are not linearly related; matrix composition simultaneously shapes vector retention, cell behavior, and the biological baseline against which gene delivery is measured.

Surface adsorption onto hard-printed scaffolds introduces a mechanistically different constraint. Lyophilized PEI-pDNA polyplexes deposited onto 3D-printed CaP cement scaffolds produced detectable reporter expression from day 2 to day 8 *in vitro*, but lattice geometries yielded substantially lower transfection efficiency than disc geometries [94]. The mechanism was adsorptive loss. Greater exposed surface area drove polyplex binding to the charged mineral surface, removing vector from solution before cellular internalization could occur [94]. Scaffold geometry is therefore an active determinant of nucleic acid bioavailability, and mineral-rich matrices require deliberate vector selection to avoid this loss, a constraint uncharacterized for most clinical-grade CaP formulations [94].

Fabrication processing is not a neutral step preceding GAM function. It encodes retention behavior directly. In collagen-based systems, freeze-drying creates the porous architecture required for nanomedicine loading and retention, while DHT or EDC/NHS crosslinking stabilizes the scaffold and modulates release behavior before cargo incorporation [19], [99], [162]. In ceramic systems, lyophilization is used to apply polyplex coatings, while cryoprotectants help preserve vector stability during freezing [94]. As a result, gene release and transfection efficiency depend on the combined effects of vector properties, scaffold composition, and fabrication method rather than any single factor alone [25].

### 6.3 Substrate-mediated transfection and the 3D microenvironmental context

Vector design sets the ceiling on transfection potential. The scaffold microenvironment determines how much of that potential is realized. 3D confinement alters cell morphology, cytoskeletal organization, and the spatial availability of polyplex cargo relative to the cell surface, effects that monolayer models cannot capture [163]. MSCs transfected within collagen-alginate hydrogels showed greater marker expression than cells transfected in two dimensions before encapsulation. This result is attributable to 3D-specific changes in polyplex spatial availability and cytoskeletal configuration rather than vector identity [164]. Within scaffold systems, expression durations consistently exceed those achievable in monolayer culture, a gap that widens with matrix crosslink density [99].

Scaffold surface chemistry can strongly influence how cells internalize polyplexes and, consequently, the efficiency of gene delivery [165], [166]. MSCs cultured on fibronectin (FN)-coated surfaces showed higher transgene expression than uncoated controls, whereas collagen I coatings reduced expression [165]. Inhibition studies identified clathrin-mediated endocytosis as the primary uptake pathway responsible for the enhanced transfection observed on FN-coated surfaces [165]. These findings suggest that scaffold coatings can regulate transfection efficiency by controlling cellular uptake of gene delivery vectors [165], [166].

Matrix stiffness, like surface chemistry, influences gene delivery efficiency. In FN-conjugated PEGDA hydrogels, increasing stiffness enhanced polyplex uptake and BMP-2 expression across multiple cell types, including BMSCs [167]. Similarly, stiffer collagen–alginate hydrogels promoted greater nanocomplex uptake and sustained SOX9 expression, an effect linked to cytoskeletal signaling and intracellular transport [164]. These findings show that both stiffness and surface biochemistry are co-equal design parameters; rational GAM engineering must treat the cell-matrix interface as an active determinant of gene transfer, not a structural backdrop [164], [166].

### 6.4 Osteogenic and angiogenic gene targets for bone regeneration

Among all osteogenic gene targets tested in GAM systems, BMP-2 has produced the most consistent evidence of *in vivo* bone formation across multiple cargo formats and scaffold compositions. Promoter engineering amplifies this further. A BMP-2-Advanced plasmid, incorporating a truncated intron sequence to improve gene expression, was delivered via chitosan nanoparticles within a collagen-HA scaffold. This construct enhanced calcium deposition by MSCs *in vitro* and generated greater bone formation than a conventional BMP-2 plasmid scaffold *in vivo*, indicating accelerated osteogenic differentiation [162]. Similarly co-delivery of pBMP-2 and pVEGF within a collagen-HA scaffold accelerated calvarial defect repair beyond what either scaffold architecture or single-factor delivery achieved in isolation [99].

The shift from plasmid to cmRNA sharpens BMP-2 delivery with enhanced efficacy and safety. Co-delivery of BMP-2 and VEGF-A cmRNA in collagen scaffolds promoted both osteogenesis and angiogenesis in a rat calvarial defect model, outperforming single-factor and recombinant protein groups in ALP activity and calcium nodule formation [160]. In critical-sized femoral defects, BMP-2 cmRNA achieved complete defect bridging at a 50 μg dose without the excessive bone formation and delayed mechanical recovery associated with recombinant BMP-2 [23]. Nucleic acid-mediated delivery can therefore match recombinant protein outcomes at substantially lower doses, though optimal cmRNA dose, carrier formulation, and defect geometry remain incompletely characterized across preclinical models [23].

VEGF as an angiogenic target contributes to defect repair through mechanisms that extend beyond neovascularization alone. Non-viral dual delivery of pBMP-2 and pVEGF via nanoHA particles within a collagen-nHA scaffold was tested in a rat cranial defect model. Bone formation increased 36-fold over empty defect controls at 4 weeks [168]. The dual-gene scaffold generated more vessels than any other experimental group, with greater mineralization than single-gene and gene-free scaffolds confirmed *in vitro* [168]. The high-expression PEI dual scaffold showed paradoxically lower mineralization, an outcome that dissociates transgene output level from osteogenic efficacy [168]. VEGF therefore functions as a mechanistically coupled co-target. Its osteogenic contribution depends on coordinated BMP-2 co-expression, not on expression magnitude alone.

MiRNA-based targeting extends the available gene target repertoire by engaging regulatory networks rather than delivering single effector proteins. A RALA-miR-26a nanoparticle-activated collagen-nHA scaffold produced up to 84-fold miR-26a overexpression in human MSCs, with corresponding increases in VEGF mRNA and protein secretion, ALP activity, and calcium deposition *in vitro* [169]. Implanted cell-free into a 7 mm calvarial defect for 8 weeks, the scaffold achieved 1.9-fold greater bone volume and 55% higher bone mineral density than a miR-free scaffold. Vascularization also trended higher [169]. Across cargo types and scaffold systems, osteogenic and angiogenic gene targets consistently produce superior outcomes when downstream signals are coordinated spatially and temporally within the matrix, though the optimal coordination window remains experimentally undefined.

### 6.5 Multi-factor and sequential delivery GAMs

Single-target gene delivery activates one arm of the repair cascade. Section 3.5 establishes osteogenic and angiogenic signals must be coordinated rather than delivered independently. Multi-factor GAMs operationalize that coordination directly, co-delivering two or more nucleic acid cargoes within a single construct, each targeting a distinct cellular program. The design logic rests on whether simultaneous or sequential release better recapitulates the biology of repair, a distinction with measurable consequences for both transfection efficiency and downstream osteogenic output.

Conventional composite scaffolds achieve sequential release through physical release kinetics, not gene expression. Section 3 describes a staged VEGF/FGF-2-then-BMP-2 scaffold that outperformed simultaneous co-delivery through this same mechanism [75]. Delivery intervals, however, remain scaffold- and defect-specific rather than fixed [76]. A PLGA microsphere/PPF composite releasing VEGF and BMP-2 sequentially enhanced bone formation at an ectopic site but produced only suboptimal formation in an orthotopic femoral defect [170]. A release profile tuned for one site does not transfer predictably to another.

GAM regulation works differently. Expression duration depends on transcriptional and translational kinetics inside infiltrating host cells. Matrix degradation rate governs duration as well, not a pre-set diffusion or hydrolysis curve. This grants GAMs a theoretical capacity against a protein-based sequential system in the same defect model.

Delivery sequence is a deterministic variable in multi-factor GAM design. Simultaneous delivery of PDGF and BMP-2 can produce antagonistic effects on tubule formation [171]. Sequential delivery yields superior outcomes, namely PDGF-B first to recruit progenitors via mitogenesis and chemotaxis, followed by sustained BMP-2 to drive osteogenic differentiation [171]. An intraoperatively bioprinted collagen-chitosan-nanoHA GAM encoding both targets achieved approximately 90% bone coverage area and 7-fold higher RUNX2 intensity than empty defects at 6 weeks in a rat calvarial model [171]. Sequential pDNA co-delivery combined with intraoperative fabrication can therefore eliminate pre-surgical scaffold preparation while preserving ordered growth factor signaling, though performance outside rat calvarial geometry remains untested.

Simultaneous mRNA co-delivery offers a mechanistically distinct approach. VEGF mRNA exerts a direct osteoblastic function, upregulating OPN, OCN, and Osterix in primary osteoblast-like cells independently of its angiogenic role [172]. Despite protein translation lasting only approximately 4 days, downstream osteogenic marker expression persisted to day 11 [172]. Runx2 and VEGF mRNA coadministration via polyplex nanomicelles into rat mandibular defects produced greater bone volume, BV/TV ratio, and bone mineral content than either mRNA alone, with CD31-positive vascularization indicating additive osteogenic-angiogenic coupling [172]. Stromal cell-derived factor (SDF-1α) as an accessory angiogenic cargo further extends this logic; scaffold-mediated pSDF-1α transfection sustains chemokine secretion and induces endothelial tubulogenesis at levels comparable to a VEGF-positive control *in vitro* [173]. Multi-factor GAMs aligning osteogenic commitment with vascular priming consistently outperform single-target constructs, though direct head-to-head comparisons across cargo formats remain absent from the literature.

The convergence of scaffold-mediated retention, microenvironmentally regulated transfection, and coordinated multi-target signaling represents the current design frontier of GAM engineering. Their integration into a clinically viable construct remains incomplete; manufacturing scalability, immune response to nucleic acid cargo, and performance in human-scale defects are each unresolved at the preclinical-to-clinical interface. Three interdependent engineering problems define this gap. Adsorptive loss onto charged CaP surfaces removes polyplex cargo before cellular internalization can occur, a constraint that worsens nonlinearly as scaffold surface area increases [94]. Sequential expression windows across multiple cargo formats are currently approximated through differential loading density rather than programmed through material design, a substitution that no published construct has yet overcome. Transfection efficiency in 3D constructs declines sharply with infiltration depth, leaving defect interiors at clinically relevant volumes undertreated regardless of surface-level performance [25]. How the intellectual property landscape has responded to these engineering constraints, and where it has not, is the subject of Section 7.

Immune timing remains uncharacterized in GAM design, unlike the osteogenic-angiogenic coordination established in Section 3.5. Macrophage polarization follows its own schedule, an early M1 phase giving way to a pro-regenerative M2 phase [122]. This transition shapes the local osteogenic and angiogenic potential. Cell-based GAMs skew polarization toward M2 more effectively than recombinant protein controls [129]. What remains undefined is the coordination itself: whether immune-modulating cues should precede, coincide with, or follow osteogenic and angiogenic signals. No GAM study has tested that ordering directly.

## 7 Patent landscape and industrial translation

Scientific output in GAM research has consistently outpaced intellectual property (IP) activity. Most foundational constructs remain unpatented or covered only through academic institutional filings, while large orthobiologics manufacturers hold dominant positions in protein-based BMP delivery that constrain the commercial entry space [15], [25]. The vast majority of GAM platforms demonstrating *in vivo* efficacy remain unpatented and outside regulatory filing pathways [3]. Three patent clusters define the current landscape, namely scaffold-vector architecture, nucleic acid cargo formulations, and personalized implant manufacturing, the last being the only segment with a confirmed clinical translation record [174]. Key granted patents and applications across these clusters are summarized in Table 3.

**Table 3:** Key patents and patent applications defining the current intellectual property landscape for gene-activated matrices in bone tissue engineering.

Patent/App. No.	Assignee	Filing Year	Grant Year	Cargo/Vector	Platform Cluster	Broadest Independent Claim (Summary)	Est. Expiry	Gov. Rights	Ref.
US 5,631,142	Genetics Institute, Inc. (Wyeth/Pfizer lineage)	1993	1997	Recombinant BMP-2 protein produced from transformed cells	Protein composition (foundational)	Purified BMP-2 composition produced from cells transformed with a defined BMP-2 DNA sequence (nucleotides #356–#1543, Fig. 2); optional matrix carrier (HA, collagen, TCP, PLA). Protein expression claim; not a gene-delivery or nucleic acid cargo claim.	Expired	None	[27]
US 8,961,999 B2	Baylor College of Medicine & William Marsh Rice University	2010	2015	Viral BMP-encoding nucleic acid; *ex vivo* transduced cells in PEG-DA hydrogel microspheres	Scaffold-vector architecture (viral/hydrogel microsphere)	Viral BMP gene-transduced cells (≤100 cells/microsphere) encapsulated in PEG-DA hydrogel microspheres fabricated by emulsification; delivered to targeted bone site. Cell-based gene therapy claim; not a direct scaffold or RNA delivery claim.	∼2030 (expired – fee-related)	Yes (DARPA)	[28]
US 11,406,739 B2	“NextGen” Co. Ltd. (Moscow, Russian Federation; SME)	2015 (PCT priority); filed 2017	2022	pDNA (VEGF, SDF-1, BMP, or IGF-1); surface-loaded on 3D-printed CaP scaffold	Personalized implant manufacturing	Method for CT-modeled, patient-specific 3D-printed CaP scaffold directly activated with surface-localized pDNA (50–500 μg/g); final implant geometry fits defect walls within ≤1 mm. Central claims cover activation during or after printing; pDNA encoding at least one of VEGF, SDF-1, BMP, or IGF-1.	∼Oct. 2035	None	[29]
US 12,016,779 B2	University of Southern California	2017 (provisional 2016)	2024	Lentiviral BMP-2 transcriptional activation system; cells cultured on CaP scaffold exterior	Scaffold-vector architecture (regional gene therapy)	Method combining a custom 3D CaP tube-scaffold (inner surface matched to healthy bone) placed in defect, then *ex vivo* lentiviral BMP-2 transduction of cells and *in vitro* culture on scaffold exterior; scaffold placement precedes transduction (as recited in claim 1). Method claims only; BMP-2 in independent claim 1.	∼Sep. 2037⁎	Yes (NIH, NSF)	[30]
US 12,133,933 B2	University of Southern California	2020 (continuation/related application in the USC regional gene therapy scaffold family (including US 12,016,779 B2))	2024	Lentiviral vector system (trans-activator + growth factor vector, MOI 3 each); broader cell sources	Scaffold-vector architecture (regional gene therapy; continuation)	Continuation broadening cell sources to MSCs, bone marrow cells, fibroblasts, adipose-derived, umbilical cord, and muscle cells (MOI 3 per vector); cells loaded on inner scaffold surface; scaffold stabilized with biocompatible plate or rod post-placement.	Term governed by continuation chain and terminal disclaimer	Yes (NIH, NSF)	[31]
US 12,263,229 B2 (published as App. 20180214572 A1)	Ethris GmbH (Planegg, Germany; SME)	2015 (PCT/EP priority 2014)	2025	BMP-encoding RNA + oligo(alkylene amine) cationic lipidoid (Formula IV); chemically modified (m5C/s2U) in dependent claims	Nucleic acid cargo formulation (cmRNA + lipidoid)	Pharmaceutical composition of BMP-encoding RNA delivered via a defined cationic lipidoid (Formula IV oligo[alkylene amine]); claim not vehicle-agnostic. Dependent claims specify BMP-2/BMP-7, m5C/s2U chemical modifications, scaffold/matrix delivery (collagen, fibrin), and *ex vivo* MSC/adipose-derived cell use.	∼Nov. 2035⁎	None	[32]
WO 2019/246203 A1; US 12,357,580 B2	Board of Regents, University of Texas System (Siegwart lab, UT Southwestern)	2019	WO 2019; US granted 2025	Ionizable LNP; nucleic acids >80 nt (broad payload; mRNA and long nucleic acids)	Nucleic acid cargo formulation (ionizable LNP platform)	Broad LNP composition for nucleic acids >80 nt; defined ionizable lipid, phospholipid, steroid, and PEGylated lipid molar ratios. Independent claim is not bone-specific and does not require BMP cargo; SORT organ-selective delivery concept is described in related Siegwart literature but is not recited in claim 1 of this WO/US publication.	∼Jun. 2039	Yes (DoD)	[33]
WO 2023/220734 A2 (US national phase pending: US 2025/0319036 A1)	Trustees of the University of Pennsylvania (Mitchell, Xue, Weissman, Alameh)	2023	PCT published 2023; US national phase pending	Bisphosphonate-lipid LNP (Formula I BP-lipid); nucleic acid cargo (mRNA, siRNA, CRISPR-Cas9; bone-targeted in dependent claims)	Nucleic acid cargo formulation (bone-targeted BP-LNP)	LNP composition incorporating a bisphosphonate-containing lipid compound (Formula I) with neutral phospholipid, cholesterol, and PEGylated lipid; composition claim agnostic to nucleic acid cargo in claim 1. Bone/bone marrow cell targeting and specific nucleic acid types appear in dependent and method claims.	∼May 2043 (if granted)	Yes (NIH)	[34]

Table 3. Platform clusters correspond to the three patent families discussed in Section 7. Expiry estimates are nominal from earliest effective filing date.

⁎ Denotes patents subject to terminal disclaimer; estimates do not account for patent-term adjustments, fee lapses, or national-phase variations. Government rights reflect declared U.S. federal funding as stated on the patent face page. App., application; BP-LNP, bisphosphonate lipid nanoparticle; DoD, Department of Defense; LNP, lipid nanoparticle; MOI, multiplicity of infection; NIH, National Institutes of Health; NSF, National Science Foundation; PEG-DA, poly(ethylene glycol) diacrylate; PCT, Patent Cooperation Treaty; Ref., reference number used in manuscript text; SME, small-to-medium enterprise; TCP, tricalcium phosphate; SORT, selective organ targeting (described in related Siegwart literature; not recited in claim 1 of WO 2019/246203 A1 or US 12,357,580 B2).

### 7.1 Foundational patents: GAM architecture and non-viral delivery

Early patents in bone GAM development focussed on scaffold design rather than specific therapeutic agents or drug formulations. Their key innovation was integrating nucleic acid delivery directly into a 3D scaffold, distinguishing GAMs from conventional local gene therapy. A representative example is US 11,406,739 B2 [29], which claims a method for manufacturing a patient-specific CaP scaffold by CT-guided defect modelling and direct 3D printing. The scaffold surface is activated with pDNA encoding VEGF, SDF-1, BMP, or IGF-1, at a concentration of 50–500 μg per gram of scaffold. The implant is designed to fit defect geometry within 1 mm; and nucleic acid incorporation is permitted during or after printing. Notably, the patent does not claim a separate delivery carrier the scaffold surface itself is the gene delivery interface.

Claim 1 of US 11,406,739 does not require simultaneous delivery of all 4 genes. Rather, it covers scaffolds functionalized with pDNA encoding at least one of VEGF, SDF-1, BMP, or IGF-1. These 4 gene targets are not interchangeable; each encodes a mechanistically distinct phase of repair. VEGF drives neovascularization at the defect margin, a prerequisite for osteoprogenitor survival in avascular scaffold interiors [99]. SDF-1 acts upstream of vessel formation, recruiting CXCR4-expressing mesenchymal progenitors to the implant surface before matrix-directed differentiation begins. BMP encodes the osteoinductive signal that commits recruited progenitors to an osteoblast lineage, the therapeutic core of every clinical BMP application [133]. IGF-1 sustains mitogenic and anti-apoptotic signaling during the consolidation phase, extending the window of active bone deposition. These four targets span vascularization, progenitor recruitment, osteoinduction, and consolidation, four non-overlapping phases of repair, so a single-gene claim under this patent can cover any one phase without requiring proof of the others. Notably, the claim does not include FGF-2, which regulates progenitor proliferation and angiogenic crosstalk, and miRNA-based cargo classes, which modulate endogenous osteogenic networks post-transcriptionally, appear nowhere in the independent claim [25], [169]. Both have experimental support in scaffold-integrated delivery formats. Neither is protected.

This design principle was established experimentally before the patent was filed. In substrate-mediated transfection, pDNA adsorbed or tethered to a scaffold surface is transferred to adhering cells in situ, enabling sustained local gene expression without systemic vector distribution [19]. The scaffold thus acts not merely as a structural template but as a transfection interface, a distinction the granted claim encodes geometrically rather than biochemically. CaP is mechanistically suited to this role, as CaP scaffold surfaces adsorb pDNA-containing complexes directly, positioning the nucleic acid cargo at the cell-material interface for localized transfer [94].

The sole completed clinical trial for a bone GAM used this architecture directly. A naked plasmid construct surface-loaded onto a CaP scaffold was implanted in 20 maxillofacial patients, with no adverse events reported over 30 months of follow-up [174]. Although the trial was open-label, non-randomized, and anatomically restricted to maxillofacial defects, it remains the only human safety study of a scaffold-surface pDNA GAM. Notably, the assignee of US 11,406,739 B2 [29] is the same Russian biotechnology company or small and medium-sized enterprise (SME) whose research group conducted that trial [15], [174]. Patent protection in this cluster therefore covers the one GAM architecture with clinical safety data, a rare convergence of IP and clinical validation. By contrast, most GAM platforms remain at an early stage of development, with limited large-animal validation, no randomized clinical efficacy studies, and unresolved manufacturing challenges [3]. That evidentiary gap defines the commercial value of US 11,406,739 B2 and marks the IP white space surrounding it; no competing granted patent covers scaffold-surface pDNA delivery at human-scale defect geometry [3].

US 11,406,739 B2 claim 1 is limited to patient-specific, CT-guided 3D-printed CaP scaffolds carrying surface-bound pDNA. Alternative GAM formats, including hydrogel, electrospun polymer, and injectable ceramics, along with systems delivering FGF-2, miRNA mimics, antagomirs, or mRNA, fall outside its scope [25]. A standardized off-the-shelf CaP scaffold carrying the same gene targets is likewise excluded, because patient-specific fabrication is a required claim element [29].

### 7.2 Viral vector delivery patents

Viral vector IP in bone repair is concentrated in two institutions. Both institutions organize claims around the same therapeutic logic, namely *ex vivo* transduction of osteogenic cells followed by delivery on a 3D scaffold. The USC/Lieberman group holds the most clinically proximate grants in this cluster. US 12,016,779 B2 [30] claims a method combining a custom 3D CaP tube-scaffold with lentiviral BMP-2 transduction of cells cultured on its exterior surface. The inner scaffold surface must match the geometry of surrounding healthy bone. Scaffold placement in the defect is required before transduction is performed. The independent claim is not vector-agnostic, requiring a lentiviral transcriptional activation system with BMP-2 specified in claim 1 rather than deferred to dependent claims. Method claims only were granted. Architecture, not formulation, was the granted right.

The continuation, US 12,133,933 B2 [31], broadens the cell-source language to include MSC, bone marrow cells, fibroblasts, adipose-derived cells, umbilical cord cells, and muscle cells. Each cell type is transduced at a multiplicity of infection of 3 per vector component. Loading is specified on the inner rather than exterior scaffold surface. This continuation extends enforceability across a wider donor-cell landscape while the parent patent holds the narrower BMP-2 position [15]. Both grants carry National Institutes of Health (NIH) and National Science Foundation (NSF) government rights, a constraint that limits licensing freedom for commercial assignees.

The earlier Baylor/Rice grant, US 8,961,999 B2 [28], claimed viral BMP gene-transduced cells encapsulated in poly(ethylene glycol) diacrylate hydrogel microspheres and is now expired-fee-related, removing it as an active blocking patent [15]. Its expiry narrows the prior-art barrier for new hydrogel-based viral delivery filings. The lentiviral specificity of the USC claims reflects a documented biological rationale. AAV vectors transduce human MSCs at efficiencies too low for reliable BMP-2 output in *ex vivo* bone repair protocols, pushing regional gene therapy development toward lentiviral platforms despite their higher genotoxicity profile [18]. The donor and processing variability described in Section 8.1 is not addressed at the manufacturing-process level by any granted patent [175].

US 12,016,779 B2 claim 1 is method-specific and narrower than its therapeutic ambition suggests. The lentiviral transcriptional activation system is required; alternative viral and non-viral vectors fall outside the claim. BMP-2 is specified in claim 1 rather than deferred to dependent claims, excluding other osteogenic transgenes at the independent claim level. The continuation US 12,133,933 B2 broadens enforceability across donor-cell types but does not extend cargo beyond the lentiviral format. Any non-viral, AAV-based, or mRNA-encoding cell-loaded scaffold approach operates outside both grants, as does any *ex vivo* protocol omitting the recited scaffold-placement-before-transduction sequence [30], [31].

### 7.3 Nucleic acid cargo IP: mRNA, miRNA, and CRISPR-based systems

The nucleic acid cargo patent cluster is the most active and least settled segment of the GAM IP landscape. Claims in this space do not center on scaffold geometry but on the chemical identity of the delivery vehicle and the modification state of the cargo. US 12,263,229 B2 [32], granted to Ethris GmbH in April 2025 after a decade-long prosecution, claims a pharmaceutical composition of BMP-encoding RNA delivered via a defined oligo(alkylene amine) cationic lipidoid of Formula IV. The claim is not vehicle-agnostic. Scaffold and matrix delivery, cmRNA with 25% 5-methylcytidine and 25% 2-thiouridine substitutions, and *ex vivo* MSC use appear only in dependent claims. The biological rationale for this cargo class is experimentally grounded. Chemically modified BMP-2 mRNA induced osteogenic differentiation in stem cells and tissue explants and accelerated bone healing in rats, establishing cmRNA as a viable non-integrating alternative to plasmid and viral constructs [21]. Optimized cmRNA encoding BMP-2 subsequently healed large osseous segmental defects more efficiently than recombinant protein, though the collagen delivery matrix was not engineered for mRNA protection or controlled release [23].

The UPenn bisphosphonate-LNP application, WO 2023/220734 A2 [34], takes a complementary approach. Its independent claim covers an LNP composition incorporating a bisphosphonate-containing lipid of Formula I with defined molar ratios of neutral phospholipid, cholesterol, and PEGylated lipid. Bone and bone marrow cell targeting appear in dependent and method claims rather than in claim 1. The experimental basis for this targeting strategy is well established, as bisphosphonate-functionalized lipid-like materials selectively delivered mRNA to the bone microenvironment *in vivo*, with uptake confirmed in osteoblasts and osteoclasts [156]. The UT/Siegwart platform, WO 2019/246203 A1 [33], issued in the US as 12,357,580 B2 [176], covers a broad ionizable LNP composition for nucleic acids exceeding 80 nucleotides. The independent claim is not bone-specific, and the selective organ targeting concept associated with this group's broader work is not recited in claim 1.

To date, no granted patent has been identified that specifically claims scaffold-integrated Clustered Regularly Interspaced Short Palindromic Repeats (CRISPR) delivery for bone regeneration. The two experimental strategies differ mechanistically in ways that matter for both safety assessment and IP positioning. dCas9-VPR, a nuclease-inactive CRISPR activator, enhances expression of endogenous genes without cutting genomic DNA, reducing the risk of permanent off-target mutations [177]. Non-viral dCas9-VPR co-activating VEGF-A and TGF-β1 within a dual-crosslinked hydrogel produced greater osteogenic commitment than either target alone [177]. Cas9 immunogenicity remains a potential safety concern regardless of whether the protein is nuclease-active or nuclease-inactive. Non-viral ribonucleoprotein delivery within 3D scaffolds has not reached transfection efficiencies comparable to plasmid or mRNA cargo in equivalent formats [177]. Nuclease-active Cas9 was used to knock out Noggin, a BMP antagonist, thereby enhancing BMP-2-mediated mineralization on silk scaffolds without the need for additional growth factors. Permanent genomic editing in primary progenitors, however, carries potential genotoxic risks that remain insufficiently characterized, and off-target assessment was absent from that study [178]. Neither system has entered large-animal validation, and delivery efficiency in scaffold-integrated formats remains uncharacterized for both.

The three nucleic acid cargo grants occupy non-overlapping claim spaces. US 12,263,229 B2 is limited to a specific oligo(alkylene amine) lipidoid structure, excluding alternative lipid chemistries, polymeric carriers, and scaffold-integrated delivery systems from its independent claim scope [32]. WO 2023/220734 A2 protects a bisphosphonate-lipid LNP composition, with bone targeting and specific cargo types appear only in dependent claims [34]. US 12,357,580 B2 has the broadest nucleic acids coverage encompasses cargoes longer than 80 nucleotides, but does not specifically claim bone regeneration at the independent claim level [33], [176]. Notably, none of these patents covers scaffold-embedded delivery of any of their respective formulations.

### 7.4 Commercial translation and patent holders: industry vs. academic IP

Academic institutions dominate the current GAM and gene-activated scaffold IP estate. Of the patents identified across the three platform clusters, the majority list universities or public research bodies as assignees. Federal funding declarations from NIH, NSF, or DoD appear on the face pages of grants from USC [30], [31], UT Southwestern [33], and UPenn [34]. Government rights clauses in these patents restrict certain licensing practices and complicate exclusive commercialization agreements, a structural challenge that bears directly on the academic startup pathways discussed by Takahashi et al. [179]. The lone SME-held scaffold patent in this set is US 11,406,739 B2, filed by NextGen Ltd. of Moscow. It represents a private-company model in which a single proprietary platform was advanced from filing through compassionate-use clinical application, without academic licensing intermediaries [29], [174].

Government rights on the USC and UPenn patents operate on two distinct tracks. The first is automatic. Under the Bayh-Dole Act, every subject invention arising from federal funding carries a nonexclusive, nontransferable, irrevocable, paid-up license permitting the government to practice the patent for its own use. This right attaches regardless of any exclusive license subsequently granted to a commercial partner [180]. The second track is contingent. The funding agency may compel licensing to third parties under four statutory conditions codified in 35 U S C. § 203, the most commercially relevant being failure to achieve practical application, defined as making the invention available on reasonable terms [180], [181]. No federal agency has exercised this authority in over forty years, and NIH has rejected every march-in petition submitted to date [181]. An orthopedic licensee acquiring exclusive rights to US 12,016,779 B2 [30] or US 12,133,933 B2 [31] from USC, or to WO 2023/220734 A2 [34] from UPenn, does not face imminent compulsory licensing. It is a structural ceiling on exclusivity that cannot be contracted away and that activates if commercialization stalls.

The Ethris GmbH portfolio occupies a distinct position. US 12,263,229 B2 [32] originates from academic inventors, namely Balmayor, Rudolph, and Plank, but is assigned to a purpose-built biotech entity. This structure avoids the government-rights constraints attached to NIH- or NSF-funded academic filings [32]. The spinout model assigns platform IP to a purpose-built commercial entity, developed through private investment and venture capital rounds. This is a documented pathway for advancing regenerative medicine technologies toward clinical translation [179]. The legacy corporate estate is represented by the expired US 5,631,142 [27], a Genetics Institute filing later acquired through the Wyeth/Pfizer chain. This patent established foundational BMP protein IP underpinning INFUSE Bone Graft, but provides no live blocking protection against nucleic acid-based delivery approaches [27].

Clinical translation data remain scarce. The NextGen Gene-Activated Implant (GAI) is the only patented GAM construct with published human outcome data, drawn from a small compassionate-use cohort [174]. The USC/Lieberman platform [30], [31] and Ethris RNA formulation [32] remain in preclinical or early investigational phases. Wilkinson et al. assessed orthopedic gene therapy constructs against a technology readiness framework and concluded that methodological inconsistency and insufficient safety data in preclinical models are the principal obstacles to IND-stage progression [29]. Manufacturing scalability and immune response to vector or nucleic acid cargo compound these barriers at the regulatory interface [175]. Patent protection precedes clinical viability; it does not produce it.

### 7.5 Patent gaps, expiries, and white-space opportunities

The current patent estate leaves substantial design space unprotected. Expired and lapsed grants define the clearest entry points. US 5,631,142 has been expired for decades, and US 8,961,999 B2 is listed as expired-fee-related [27], [28]. Neither poses a freedom-to-operate barrier for nucleic acid-based GAM development. The expiry of protein-era BMP IP removes the most historically restrictive layer of the orthobiologics estate and opens composition-of-matter space for any construct that delivers osteogenic transgenes rather than recombinant protein.

Among active grants, the earliest expiries fall in 2035. US 11,406,739 B2 (NextGen) and US 12,263,229 B2 (Ethris) both reach nominal term around that date [29], [32]. Neither is broad enough to block the full GAM design space. The NextGen patent is constrained to patient-specific CaP scaffolds activated with surface-localized pDNA at defined nucleic acid concentrations; constructs using hydrogel carriers, electrospun matrices, or injectable depots fall outside claim 1. The Ethris grant requires a specific oligo(alkylene amine) cationic lipidoid; alternative ionizable lipid chemistries are not captured. Both represent narrow platform claims, not field-wide exclusivities.

The most consequential white spaces are architectural. No granted patent covers non-viral delivery of mRNA within a 3D CaP or composite scaffold, the combination that most closely describes current GAM design priorities [3], [25]. The USC/Lieberman patents, US 12,016,779 B2 and its continuation US 12,133,933 B2, address lentiviral cell-based regional gene therapy on ceramic scaffolds [30], [31], and the UPenn and UT LNP patents address systemic or injectable nucleic acid delivery [33], [34], [176]. The intersection of scaffold-embedded non-viral mRNA delivery, however, remains unclaimed. Additional unclaimed areas include sustained-release formulations, stimulus-responsive vector release, and miRNA-activated constructs within porous scaffolds [25]. With key patents beginning to expire around 2035 and limited filing activity in these areas, substantial opportunities for future IP protection remain.

Two cargo targets with scaffold-integrated experimental records sit entirely outside the current patent estate. SDF-1α delivered as pDNA within a chitosan-nanoparticle collagen-HA scaffold promoted recruitment of CXCR4-expressing mesenchymal progenitors and achieved complete defect bridging in a rat calvarial model. Osteogenic markers including Runx2, Dlx5, and Sp7 showed early upregulation [182]. The mechanism is upstream of both BMP-driven osteoinduction and VEGF-driven angiogenesis, SDF-1α acts primarily as a chemotactic recruitment signal rather than a differentiation or vascularization effector. FGF-2 occupies a similar position, supporting progenitor proliferation and angiogenic signaling, yet neither target is currently claimed as cargo in a scaffold-integrated non-viral delivery [25], [182]. Both targets are mechanistically differentiated, experimentally validated, and IP-unprotected in this delivery format.

## 8 Challenges and translational barriers

Clinical translation requirements depend on the specific design and composition of each GAM construct, and do not apply uniformly across the platform. Substituting mRNA for pDNA, or a viral vector for a polyplex, alters the stability profile, the sterilization route, the safety data package, and the regulatory classification of the finished product. Adding a cellular component changes all four again. Evidence generated for one construct therefore rarely reduces the evidentiary burden on another. The barriers below are addressed in the order a construct encounters them, from manufacture to human evidence.

### 8.1 Manufacturing, sterilization, and product class

Manufacturing burden separates the two GAM product classes more sharply than any biological property. Acellular constructs combine a scaffold with nucleic acid or vector and contain no living component, so they can be produced in advance and implanted in a single operation. Control rests on the fabrication route. Scaffold architecture, cargo loading efficiency, and dose uniformity across a batch must fall within defined release specifications, and the plasmid requires a pharmaceutical-grade supply chain [174]. The maxillofacial gene-activated bone substitute that reached clinical use was supplied in glass vials held at 2 to 6 °C, with plasmid DNA obtained from an external manufacturer [174]. What these constructs concede is biological, since transfection relies on host cells migrating into the implant, a population often depleted at sites of massive bone loss [15].

Cell-based designs invert this profile. Cells are commonly harvested from the patient, transduced *ex vivo*, expanded, and seeded onto the scaffold, which delivers a committed osteogenic population rather than relying on recruitment [15]. Every step adds a constraint. Donor cells vary in yield, growth rate, and receptiveness to modification, producing inter-patient and batch-to-batch variability [175]. Potency testing, cryopreservation or fresh-product logistics, and qualified good manufacturing practice (GMP) clean rooms are all required, with per-patient manufacturing costs estimated at $100,000 to $300,000 or more [175]. Multi-week processing also sits poorly with acute trauma, although same-day protocols shorten the interval by implanting fewer transduced cells [15]. Acellular GAMs are therefore more compatible with off-the-shelf use, whereas cell-based GAMs offer greater biological control at substantially higher manufacturing, release-testing, and logistical cost.

Sterility assurance applies to both classes and has no generic solution. Terminal methods are constrained by formulation-specific thermal and radiolytic sensitivity, as autoclaving can alter particle size and cargo retention in lipid-based carriers, gamma irradiation can generate radicals that damage lipids and cargo, and ethylene oxide may leave reactive residues [41]. Sterile filtration is a practical alternative, but it is itself formulation dependent. The diameters of mRNA-lipid nanoparticles overlap with the nominal 0.2 μm pore size of sterilizing-grade membranes, creating fouling and yield constraints, and filtration capacity varies with membrane pore morphology and prefilter selection [42]. Process development must therefore be product-specific rather than assumed transferable. Storage is equally formulation specific. In one mRNA-LNP model, conventional sugar cryoprotectants retained approximately 60–70% of transfection activity after four weeks at −80 °C [43]. The reviewed preclinical studies generally do not establish a validated, product-specific shelf life.

### 8.2 Regulatory classification and approval pathways

Classification is determined by the finished product and its primary mode of action, not by the technology label. In the United States, a scaffold carrying a nucleic acid or vector may be regulated as a combination product. The primary mode of action then sets the lead review center and the development requirements [44]. Where the effect is mediated by transgene expression rather than by the scaffold, the gene therapy component drives that determination.

Europe treats the components separately rather than as a single regulated entity, so device and medicinal product requirements may apply in parallel [44]. Classification therefore depends on the specific product and an acellular scaffold carrying nucleic acid requires its own assessment. Routes and expedited designations differ between the two regions and are not harmonized [3], [183].

Approved cell-scaffold products indicate what these routes demand, not how near GAMs are to satisfying them. Such products are generally judged as combination products in which the biological component is the mainstay and the device the supplement [45]. MACI is the clearest precedent. Autologous chondrocytes are seeded on a resorbable porcine type I/III collagen membrane at 500,000 to 1,000,000 cells per cm^2^, and the product has been licensed in the US since 2016 as an autologous cellularized scaffold [184]. Approved gene therapies in other indications are priced from approximately $350,000 to more than $2 million per patient, reflecting vector manufacture and small eligible populations rather than classification alone [175]. To our knowledge, no orthopedic GAM has completed Food and Drug Administration (FDA) or European Medicines Agency (EMA) marketing authorization. The maxillofacial construct that reached clinical use was authorized by the Russian Ministry of Healthcare in 2019 [174], which limits what it demonstrates about either pathway.

### 8.3 Preclinical evidence required before clinical entry

The preclinical package that precedes an investigational application is set by the vector rather than by the scaffold. A collagen matrix carrying an adenoviral PDGF-B construct was assessed in a rabbit dermal wound model over four weekly doses at 1 × 10^10^ and 1 × 10^11^ viral particles per cm^2^. Vector DNA and transgene mRNA were recovered from the wound and the draining axillary lymph nodes, but not from blood or other organs. Hematology, serum chemistry, and histopathology showed no treatment-related changes, and antibody responses developed against the vector while anti-collagen responses did not [40]. Local expression, biodistribution, systemic safety, and immunogenicity data of this kind constitute the minimum expected before first-in-human use, and integrating vectors additionally require the insertional risk assessment discussed in Section 5.1.

Bone GAMs have not assembled an equivalent package. Orthopedic gene therapy has been placed at technology readiness level (TRL) 4, below the good laboratory practice (GLP) safety studies and GMP-compliant manufacturing required for TRL 5 and clinical entry [3]. The evidence gap is not uniform across constructs. Products that have entered human use did so with vector-specific data generated for that construct alone, which is why readiness cannot be inferred from the field as a whole.

### 8.4 Human evidence and cross-disease experience

Human evidence for bone GAM remains limited to two constructs. An open-label, non-randomized maxillofacial study of 20 patients treated with a naked plasmid construct reported no adverse events at 30 months [174]. That study was confined to a single craniofacial site and was not designed to demonstrate efficacy, so it supports feasibility and preliminary safety rather than clinical validation. A larger comparison has since been reported in the spine. A single-center randomized study allocated 200 adults undergoing single-level transforaminal lumbar interbody fusion to an octacalcium phosphate scaffold carrying VEGF pDNA or to autologous bone graft [46]. Complete fusion at 12 months reached 77% with the gene-activated material and 73% with autograft (p = 0.583), with mean fusion-zone density of 617.6 ± 180.9 against 599.8 ± 181.9 Hounsfield units (p = 0.484). Revision surgery occurred in 3% and 5% of patients respectively, and no immunogenicity, excessive angiogenesis, or abnormal tissue proliferation was reported over 12 months [46].

Interpretation is constrained. The graft sat within an interbody cage under posterior instrumentation, which is not a critical-size segmental defect, and the report provides no trial registration number, sample size calculation, or prespecified non-inferiority margin. Comparable 12-month outcomes are what these data support, not equivalence or superiority.

Registered activity beyond these reports is sparse. Four bone GAM studies appear on ClinicalTrials.gov (accessed September 2026), sponsored by two companies with overlapping academic collaborators, covering maxillofacial reconstruction (NCT03076138, NCT02293031), long bone nonunion (NCT04705857), and alveolar ridge augmentation (NCT04511689). One is recorded as completed and three carry unknown status. Two of the four are observational rather than interventional. No terminated or withdrawn bone GAM trial was identified in this search.

Scaffold-mediated gene delivery has progressed further in soft tissue, and the transferable lesson is methodological. A phase 2 trial in 124 patients with diabetic neuropathic foot ulcers compared an adenoviral PDGF-B collagen gel against the collagen carrier alone and against standard care, with complete closure in 41%, 45%, and 31% of patients respectively, and none of the differences reaching significance [39]. The carrier was not inert. Collagen can retain the negatively charged vector electrostatically, and exposure to platelets after debridement may release endogenous PDGF [39]. A bone GAM trial without a scaffold-only arm therefore risks attributing to the transgene an effect the matrix produces on its own.

### 8.5 Preclinical model limitations

Small rodent models dominate GAM literature and are valuable for early proof of concept, but their size, healing biology, loading environment, and limited capacity to reproduce clinical non-union restrict what they predict about human-scale defects [3], [175]. The mismatch is mechanistically specific rather than a matter of sample size. Rat calvarial defects heal through intramembranous ossification alone, across a cortical thickness of roughly 0.5–0.8 mm, flanked by two vascular tables that supply the repair zone from both surfaces [3]. There is no axial load, no functional periosteum contributing osteoprogenitor cells, and no marrow cavity requiring vascular invasion before mineralization. Long bone segmental defects share none of these conditions. Innate immune responses to viral vectors also differ between rodent and human progenitor populations, and two gene therapy approaches validated in rats failed entirely in sheep [8].

Critical size is a property of the model rather than a fixed dimension (Table 4). No standardized threshold exists, and the value depends on the animal, bone type, defect geometry, and cause of the defect, with reported rat calvarial thresholds generally falling between 5 and 8 mm [4]. For load-bearing evaluation, a sheep femoral mid-diaphyseal full-thickness osteotomy has been proposed as a standardizable large-animal system, and no GAM construct has been tested in it at clinically relevant scale [3]. Across large-animal bone gene therapy studies, outcomes have been broadly positive but methodologically inconsistent in species, defect site, vector, cargo, dose, and fixation, which prevents cross-study synthesis and compounds the reporting limitations noted in Section 2 [3]. The preferential publication of positive findings, together with inadequate reporting of randomization, blinding, and sample-size justification in some preclinical studies, may contribute to an overestimation of GAM efficacy and limit the reliability of comparisons across studies.

**Table 4:** Reported critical-size bone defect dimensions across commonly used preclinical species and anatomical sites.

Species	Site	Reported dimension	Translational role	Ref
Rat	Calvaria	5 mm trephine defect, reported range 5–8 mm	Early screening, non-load bearing	[4]
Rat	Femur, segmental	5 mm load-bearing defect	Mechanically relevant small animal	[35]
Rabbit	Mandible	8 × 3 × 2 mm critical, 4–6 mm healed by 12 weeks	Intermediate craniofacial feasibility	[36]
Sheep	Femur, mid-diaphyseal	42 mm segmental, intramedullary fixation	Load, fixation, and scale	[37]
Large animal	Mandible, segmental or box	20 mm canine to 50 mm porcine	Surgical feasibility, longer follow-up	[38]

Table 4. Dimensions are model-specific and reflect the conditions of the cited study rather than universal thresholds. Reported values vary with species, strain, age, anatomical site, defect geometry, and fixation method.

### 8.6 Translational staging

Advancing a GAM between stages requires changing the construct, not only the model. A formulation optimized for transfection efficiency in culture is rarely the one that survives sterilization, storage, and implantation at scale, so each transition imposes its own technical adaptation (Table 5). The sharpest discontinuity falls between small and large animal work. Rodent studies can use research-grade vector and hand-assembled scaffolds, whereas large-animal work demands batch consistency, defect geometry matched to clinical scale, and an implantation method a surgeon could reproduce [3]. Few GAM programs have crossed that point, and those that have reported feasibility with variable and incomparable outcomes [175]. Table 5 therefore sets out requirements rather than a route any bone GAM has completed.

**Table 5:** Technical adaptations required of GAM at successive stages of translational development.

Stage	Purpose	Required adaptation of the construct	Ref.
In vitro	Transfection efficiency, cytotoxicity, expression kinetics	Define cargo dose, scaffold retention, and release profile	Section 6
Small animal	Initial efficacy and local safety	Include a scaffold-only control arm; justify the defect size for the specific model; report randomization and blinding	[3], [4], [39]
Large animal	Scale, fixation, load, systemic safety	Adopt a clinically relevant defect geometry and fixation method; include biodistribution and systemic safety sampling	[3], [37], [40]
Clinical entry	Safety and feasibility	Establish sterility and stability for the final formulation; meet general quality and biocompatibility standards; confirm product classification and lead review route	[41], [42], [43], [44], [45]
Comparative clinical study	Clinical benefit	Prespecify the comparator and analysis margin, use indication-relevant endpoints, and extend follow-up beyond initial union assessment	[46]

These barriers are sequential rather than parallel, and failure at one stage forecloses the next. A formulation that loses activity during sterilization never reaches a stability study. Without biodistribution data, an investigational application cannot be filed, and a trial lacking a scaffold-only arm cannot attribute its outcome to the transgene. The spinal fusion study shows that GAM can be manufactured, implanted, and followed for twelve months in 200 patients. Efficacy in a defect the host cannot bridge unaided remains undemonstrated.

## 9 Future directions and emerging technologies

Gene-activated matrices have advanced from proof-of-concept constructs to platforms capable of spatiotemporally controlled gene expression at defect sites. However, three frontiers remain underdeveloped. Precision gene network editing via mRNA and CRISPR-based tools is one, the convergence of fabrication intelligence, stimuli-responsiveness, and computational design within next-generation GAM platforms is a second, and the integration of GAMs with cell-based therapies and bioreactor systems is a third. Together, these directions define the translational frontier of GAM development.

### 9.1 mRNA-mediated and CRISPR-based gene activation strategies

Chemically modified mRNA bypasses innate immune sensing through nucleotide substitution and acts in the cytoplasm, mitigating both the nuclear entry barrier and genomic integration risk [21]. BMP-2 cmRNA healed critical-sized femoral defects faster than rhBMP-2 and without ectopic callus. However, the collagen sponge delivery matrix was not optimized for mRNA protection or controlled release, a limitation the authors identify as the principal barrier to scaling this approach [23]. BMP-2 and VEGF-A cmRNA co-delivered in a collagen scaffold outperformed either factor alone in both mineralization and vascularization [160]. Protecting cmRNA integrity and coordinating multi-factor release within a single scaffold architecture remains the unmet engineering requirement for this cargo class to move beyond isolated preclinical demonstrations [23], [160].

CRISPR-based systems regulate endogenous gene networks rather than delivering therapeutic genes directly. Non-viral dCas9-VPR delivery co-activating VEGF-A and TGF-β1 within a hydrogel scaffold produced greater defect closure and OCN expression than either target alone [177]. CRISPR/Cas9 knockout of Noggin, an endogenous BMP antagonist, amplified scaffold-driven mineralization without supplemental growth factor, though off-target editing risk in primary progenitor cells was not evaluated [178].

Specificity cannot be assumed from the absence of double-strand breaks. In 2 human cell-lines, guides designed to activate a long non-coding RNA (lncRNA) promoter also raised IL-6 by 10.3 ± 3.2-fold, and disrupting the intended recognition sites reduced on-target activation without abolishing the IL-6 response [185]. Sequence-based prediction identified no causal off-target site. The opposite result is also on record. Transcriptome-wide profiling of a dCas9 synergistic activation mediator (SAM) system directed at the human immunodeficiency virus (HIV) long terminal repeat returned profiles closely matching controls. One guide produced no significant lncRNA change and the other altered 2 lncRNA and 21 mRNAs [186]. Specificity is therefore a property of the particular guide, activator, and cell context, and has to be established experimentally rather than predicted.

Payload size constrains delivery. AAV accommodates roughly 4.7 to 5.0 kb, which a dCas9-VPR activator exceeds [187]. Dual-vector reconstitution offers one route, and an mRNA trans-splicing system restored CRISPRa activity across mouse retina, heart, liver, skeletal muscle, and lung [187]. That approach substitutes a co-delivery requirement for a packaging limit, and its performance in a scaffold-delivered construct has not been reported.

### 9.2 Fabrication, intelligence, and stimuli-responsiveness: converging frontiers in GAM engineering

Scaffold fabrication parameters directly determine gene release and biological performance. In a 3D-printed PCL scaffold system, coating material, coating concentration, and glutaraldehyde crosslinking concentration governed the release kinetics of pDNA encoding miR-200c [188]. Basic gelatin retained pDNA more effectively than collagen or acidic gelatin. The mechanism is electrostatic, since the positively charged coating binds the negatively charged cargo. Glutaraldehyde crosslinking reduced release from collagen and acidic gelatin, and increased it from basic gelatin [188]. These manufacturing variables translated directly into biological outcome as scaffolds with optimized coating upregulated RUNX2, OCN, and ALP, and enhanced bone regeneration in a rat calvarial defect [188]. Fabrication is not a neutral step preceding function. It encodes function.

Unlike conventional scaffolds that release cargo passively over time, stimuli-responsive scaffolds enable gene release in response to specific external or biological triggers [189]. A UV-cleavable PEG hydrogel demonstrated this precisely, where cholesterol-modified miR-26a was tethered via a photocleavable ester bond, confining release to UV irradiation events [190]. Approximately 70% of cargo was released within 5 min of 365 nm exposure, tunable by irradiation time and intensity [190]. In hMSCs, intracellular miR-26a peaked at day 10, suppressed a GSK3β reporter by approximately 40%, and upregulated osteogenic proteins [190]. In a rat calvarial defect, bone volume in the UV-triggered group exceeded controls, with minimal regeneration in non-irradiated condition [190]. Although UV-responsive hydrogels demonstrate precise spatiotemporal control of nucleic acid release, the limited tissue penetration of UV light restricts their clinical applicability, particularly for deep or load-bearing bone defects [190]. Broader stimuli-responsive design encompasses magnetic, ultrasonic, ROS-sensitive, and pH-responsive inputs [189], and systems that integrate multiple triggers remain unvalidated *in vivo* at clinically relevant defect scales.

Computational tools have not yet been integrated into GAM fabrication workflows, and that gap is measurable in outcome variance. AI-assisted approaches linking architecture prediction, biodegradation modeling, and additive manufacturing optimization remain constrained by data scarcity and absence of clinical-scale validation [191]. The penalty for proceeding without this guidance is evident in published constructs. An antagomiR-138-activated collagen-HA scaffold produced 30 ± 24 mm^3^ bone volume at 8 weeks in a rat femoral defect, with 12 ± 4 vessels/mm^2^ [35]. A standard deviation approaching the mean indicated that fabrication, release, and therapeutic variables are not yet jointly optimized.

### 9.3 Convergence with cell-based therapies and bioreactor systems

Incorporating living cells into GAM scaffolds shifts the platform from passive delivery into active biological programming. Rather than relying solely on vector-laden matrices to recruit endogenous progenitors, cell-based GAMs seed the defect site with pre-committed osteogenic cells that simultaneously act as local BMP-2 factories [15], [129]. This convergence introduces a third biological variable alongside scaffold architecture and gene delivery, namely the cellular component, which carries the manufacturing and regulatory constraints described in Sections 8.1, 8.2 [15], [129].

Mechanical preconditioning through perfusion bioreactors offers one strategy for driving osteogenic commitment prior to implantation, without dependence on chemical osteogenic supplements. In a 3D microporous poly(L-lactide-co-trimethylene carbonate) scaffold model, laminar flow at 0.8 and 1.6 mL/min for 8 h daily over 21 days elevated expression of Rho-associated coiled-coil-containing protein kinase 1 (ROCK1) and increased nuclear RUNX2 localization to 8.8% and 13.6% of cells respectively. Collagen I deposition, ALP activity, and calcium mineralization were also enhanced relative to static controls [192]. The mechanotransductive pathway, linking cytoskeletal tension through ROCK1, a key regulator of mechanotransduction that mediates cellular responses to scaffold stiffness, to transcription factor activation, provides a chemical-free priming mechanism applicable to scaffold-seeded cell populations. The authors note, however, that optimal flow parameters remain species, donor, and scaffold-specific, and clinical translation requires further *in vivo* validation [192].

When GAMs are configured as cell-based manufacturing products, donor variability becomes a key determinant of product performance. In a comparison of 124 adipose and 63 bone marrow harvests, adipose-derived stem cells (ASCs) produced significantly higher BMP-2 output after lentiviral transduction than bone marrow-derived stem cells. ASCs also expanded more reliably in culture [193]. Donor age above 60 years reduced ASC BMP-2 output, while sex and BMI were not significant determinants, establishing age as the main source of variability in autologous manufacturing [193].

Cell-based GAMs also reprogram the early repair microenvironment. In femoral defects, lentiviral BMP-2-transduced ASC constructs generated more chondrocytes and stronger endochondral ossification signaling across BMP, FGF, and NOTCH pathways than rhBMP-2, alongside the M2-skewed macrophage response described in Section 3.5 [129]. Cell-based GAMs therefore act on three axes at once. They sustain transgene delivery, precondition cells mechanically, and skew the early immune microenvironment toward repair, a coordination no acellular construct has yet replicated.

## 10 Conclusion

Bone defects at the critical-size threshold remain limited not by scaffold design alone, but by the inability to sustain osteogenic signaling in the appropriate cell populations throughout a dynamic inflammatory and mechanically demanding healing environment. Each prior approach exposed a specific failure mode that the next was designed to correct. Gene-activated matrices were developed to address these challenges by integrating structural support with localized gene delivery. Analysis of the current patent landscape reveals that scaffold–vector combinations validated *in vivo*, particularly those accounting for immune sequencing, mechanical loading, and cell-source variability, remain largely unprotected. This asymmetry signals where engineering effort and IP strategy have diverged.

The gap between preclinical validation and clinical deployment is not narrowing at a pace that reflects mechanistic progress. No published GAM construct has cleared manufacturing scalability, regulatory classification as a combination product, and efficacy in a human-scale segmental defect simultaneously. Most evidence derives from rodent models, which support proof-of-concept but cannot reproduce the geometric, immunological, and vascular complexity of human skeletal repair. The remaining barriers are therefore less biological than translational, requiring advances in manufacturing, regulatory strategy, and clinically relevant preclinical validation before GAMs can achieve routine clinical use.

## CRediT authorship contribution statement

**Hiba Abdelnabi:** Writing – review & editing, Writing – original draft, Visualization, Data curation. **Salem Mohammed Alkaabi:** Writing – review & editing, Visualization, Data curation. **Khaled Saeed Alderei:** Writing – review & editing, Visualization, Data curation. **Ahmad Sultan Alaryani:** Writing – review & editing, Visualization, Data curation. **Sahar Mohsin:** Writing – review & editing, Writing – original draft, Supervision, Project administration, Methodology, Funding acquisition, Conceptualization.

## Declaration of generative AI and AI-assisted technologies in the manuscript preparation process

During the preparation of this work the authors used Claude Sonnet 5 (Anthropic) and ChatGPT (OpenAI) to assist with language editing and rephrasing, and to help identify and discuss candidate literature. All candidate articles were read in full and evaluated by the authors, who made all the final decisions on reference inclusion, scientific interpretation, and manuscript content. After using these tools, the authors reviewed and edited the content as needed and take full responsibility for the content of the published article. Figures were created by the authors using BioRender.com, an illustration platform that does not involve generative AI content creation.

## Declaration of competing interest

The authors declare that they have no known competing financial interests or personal relationships that could have appeared to influence the work reported in this paper.

## Data Availability

No data was used for the research described in the article.
